# The influence of zinc oxide utilization in ruminants: A systematic review and meta-analysis

**DOI:** 10.1016/j.vas.2026.100796

**Published:** 2026-08-01

**Authors:** Rahmat Hidayat, Mardiah Rahmadani, Fenda Alvionita Fhonna, Laily Rinda Ardani, Aldena Bina Salimah, Ujang Hidayat Tanuwiria, Adam Cieslak, Malgorzata Szumacher-Strabel, Zora Varadyova, Anuraga Jayanegara, Aeni Nurlatifah, Amlan Kumar Patra, Agung Irawan, Yulianri Rizki Yanza

**Affiliations:** a Department of Animal Nutrition and Feed Technology, Faculty of Animal Husbandry, Universitas Padjadjaran, Sumedang, 45363, West Java, Indonesia; b Doctoral Program of Agricultural Science, Postgraduate School, Universitas Syiah Kuala, Banda Aceh, 23111, Aceh, Indonesia; c Department of Animal Nutrition, Faculty of Animal Science, Universitas Andalas, Padang, 25163, West Sumatra, Indonesia; d Department of Animal Production, Faculty of Animal Husbandry, Universitas Padjadjaran, Sumedang, 45363, West Java, Indonesia; e Department of Animal Nutrition, Faculty of Veterinary Medicine and Animal Science, Poznan University of Life Sciences, Wolynska 33, 60637, Poznan, Poland; f Institute of Animal Physiology, Centre of Biosciences, Slovak Academy of Sciences, Kosice, Slovakia; g Department of Nutrition and Feed Technology, Faculty of Animal Science, IPB University, Bogor, 16680, West Java, Indonesia; h Department of Nutrition and Feed Technology, Faculty of Animal Science, Universitas Gadjah Mada, Sleman, 55281, Yogyakarta, Indonesia; i American Institute for Goat Research, Langston University, Langston, OK, 73050, USA; j Vocational School, Universitas Sebelas Maret, Surakarta 57126, Central Java, Indonesia

**Keywords:** Zinc oxide, Nano-zinc oxide, Ruminant nutrition, Rumen fermentation, Meta-analysis

## Abstract

Zinc (Zn) is an essential trace mineral involved in rumen microbial activity, nutrient metabolism, and animal productivity. This meta-analysis quantitatively evaluated the effects of ZnO supplementation on rumen fermentation characteristics, nutrient utilization, and productive performance in ruminants using 17 eligible studies published between 1971 and 2025. Standardized mean differences (SMD) were pooled using random-effects models. Heterogeneity was assessed using *I*² statistics, while subgroup analysis were performed according to ZnO form. Publication bias was evaluated using funnel plot inspection, Egger’s regression test, and trim-and-fill correction. ZnO supplementation significantly improved several *in vitro* fermentation parameters by reducing methane production (SMD = −1.281; 95% CI: −1.818 to −0.743; P < 0.001), increasing propionate concentration (SMD = 0.521; P = 0.026), decreasing the acetate-to-propionate ratio (SMD = −1.226; P < 0.001), and increasing metabolizable energy (SMD = 2.034; P < 0.001). *In vivo*, ZnO supplementation reduced ruminal ammonia concentration (SMD = −0.869; P < 0.001) while increasing total volatile fatty acids (SMD = 0.520; P = 0.018), dry matter digestibility (SMD = 9.944; P < 0.001), average daily gain (SMD = 0.565; P = 0.004), dry matter intake (SMD = 0.649; P = 0.020), and alkaline phosphatase activity (SMD = 0.822; P = 0.033). Subgroup analysis revealed that nZnO consistently produced larger effect sizes for methane mitigation and growth responses than ZnO. Although publication bias was detected for methane outcomes, trim-and-fill analysis confirmed the robustness of the antimethanogenic effect (adjusted SMD = −1.082; P < 0.001). These findings provide quantitative evidence supporting that the supplementation of ZnO is a feasible option to improve productivity and sustainability in ruminant feeding systems.

## Highlights

• ZnO reduced methane production and improved rumen fermentation.
• Nano-ZnO showed stronger antimethanogenic effects than ZnO.
• ZnO increased digestibility, feed intake, and daily weight gain.
• ZnO improved propionate production and metabolizable energy.
• ZnO supports sustainable and efficient ruminant production.

## Introduction

Zinc (Zn) is an essential trace element that functions as a catalytic cofactor in >300 metalloenzymes and as a structural component of zinc-finger transcription factors regulating gene expression, cell proliferation, and differentiation (Yatoo et al., 2013). Physiologically, Zn plays an essential role in immune function, milk quality, hoof and skin integrity, and reproductive performance. Subclinical deficiency can impair both cell-mediated and humoral immunity, increase susceptibility to infections, elevate somatic cell counts in milk (indicating subclinical mastitis), disrupt epithelial barriers, and lead to poor keratin formation, skin lesions, infertility, and embryonic loss (Kegley et al., 2016). In addition, because Zn is essential for enzymatic activity and protein synthesis, subclinical deficiency may reduce growth performance, including average daily gain (ADG) and feed efficiency. In pre-weaned calves, inadequate Zn status further predisposes animals to enteropathogenic infections and diarrhoea, representing a major welfare and economic concern under intensive rearing conditions (Liu et al., 2023).

Among commercially available Zn sources, zinc oxide (ZnO) remains the most widely used in ruminant rations due to its high elemental Zn concentration (approximately 80%), low cost, physical stability in premix matrices, and compatibility with standard feed manufacturing (Vigh et al., 2023). However, the ruminant digestive environment presents inherent bioavailability challenges for ZnO that do not apply to monogastric species. The alkaline rumen pH, elevated microbial sulfide concentrations, and competitive antagonism from dietary calcium, copper, and iron promote precipitation of Zn as insoluble sulfide and phytate complexes, substantially reducing the pool of soluble Zn available for post-ruminal absorption (Hosseini-Vardanjani et al., 2020). As a result, between 70 and 95% of ingested ZnO is recovered in faeces depending on dietary conditions, constituting both a bioavailability inefficiency and a source of environmental Zn loading through manure application to agricultural soils (Maqsood et al., 2022).

To address these limitations, nano-structured ZnO (nano-ZnO) has been proposed as an alternative supplement with greater specific surface area, enhanced dissolution under physiological pH, and higher bioavailability at lower total Zn doses compared with conventional ZnO (Geetha et al., 2020). Nano-ZnO also exhibits intrinsic antimicrobial activity through reactive oxygen species generation and membrane disruption, which may provide additional benefits for pathogen suppression and modulation of the gut microbiome (Zarghami et al., 2025). *In vivo* studies in ewes have shown that partial or complete substitution of conventional ZnO with nano-ZnO improved animal responses. Nano-ZnO increased dry matter intake (11%), apparent nutrient digestibility (6%), rumen antioxidant capacity (30%), and Zn concentration (20%) compared to control, without evidence of hepatotoxicity (Hosseini-Vardanjani et al., 2020). *In vitro* studies using rumen fluid incubation systems have demonstrated that both conventional and nano-ZnO influence rumen fermentation characteristics in a dose-dependent manner. For example, supplementation with nano-ZnO at 0.1% increased total volatile fatty acid (VFA) concentration by 9.4% and acetic acid concentartion ny 6.5%, while reducing methane production by 46.8% compared with the control treatment. Nano-ZnO generally produces more pronounced effects per unit of Zn due to its greater surface reactivity (Sarker et al., 2018). At supraoptimal concentrations, however, nano-ZnO has been shown to impair *in vitro* dry matter digestibility and depress gas production, suggesting a narrow optimal concentration range before pro-oxidant inhibition of cellulolytic rumen microorganisms occurs (Abdollahi et al., 2019)

Despite a growing primary literature, several critical limitations prevent clear, practice-oriented conclusions regarding Zn use in ruminant nutrition. First, existing meta-analysis either pool Zn indiscriminately with other inorganic Zn sources (Liu et al., 2023) or include it among mixed Zn treatments without source-specific disaggregation (Angeles-Hernandez et al., 2021), obscuring effect estimates that are directly relevant to nutritional formulation decisions. Second, nano-ZnO has been excluded entirely from published quantitative syntheses, despite its increasing commercial availability and distinct physicochemical behaviour. Third, the *in vitro* and *in vivo* evidence streams have never been synthesised within a unified analytical framework, limiting mechanistic interpretation of performance and fermentation responses. These gaps are particularly consequential given that the European Food Safety Authority FEEDAP Panel (2012) has recommended reductions in maximum permitted dietary Zn levels for ruminants to 150 mg/kg DM based primarily on environmental risk modeling of soil accumulation, rather than on formal dose-response evidence at the animal level. Establishing the minimum effective Zn dose that sustains animal performance and health is therefore an urgent evidence need for both nutritional and regulatory purposes.

The present study addresses these gaps through a systematic review and meta-analysis, applying random-effects models, and formal risk-of-bias assessment. The specific objectives are to systematically identify and appraise controlled *in vitro* and *in vivo* trials evaluating ZnO forms in ruminant diets and derive pooled effect size for key fermentation, performance, and health in ruminant.

## Material and methods

### Database construction and eligibility criteria

A systematic literature search was conducted in the Scopus database on 18 January 2026 to identify relevant studies examining ZnO supplementation in ruminants. The search was performed without restriction on publication year to ensure comprehensive coverage of the available literature. Scopus was selected as search platforms due to its strict indexing standards that enhance data reliability, and its ability to provide consistent metadata, minimize duplication, and support structured and reproducible literature screening, thereby strengthening methodological rigor in evidence synthesis. The search strategy within title, abstract, and keywords employed the keywords (“zinc oxide” OR “ZnO” OR “nano zinc oxide” OR “zinc oxide nanoparticle”) AND (rumen OR ruminant). No language or species filters were applied at the search stage to minimise the risk of missing relevant records; non-ruminant and non-eligible species were later excluded during screening. All potentially relevant records were subsequently imported into a reference management system for further screening.

**Fig. 1:**
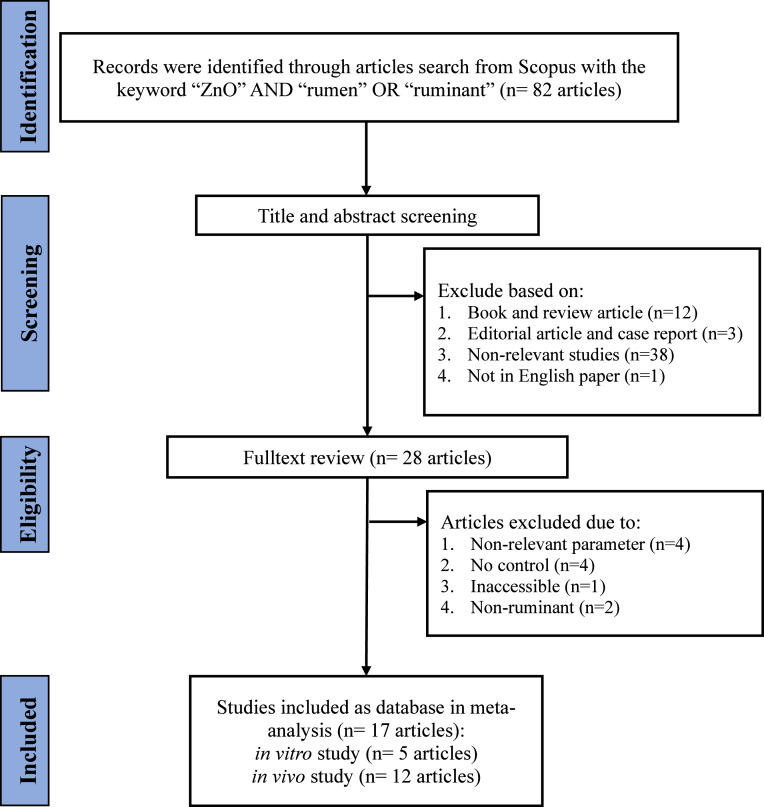
Flow charts of studies included in the meta-analysis.

An initial pool of 82 articles was identified based on title relevance. These records were then evaluated using predefined inclusion criteria aligned with the Preferred Reporting Items for Systematic Reviews and Meta-Analysis (PRISMA) guidelines to ensure methodological reproducibility (Liberati et al., 2009). Studies were considered eligible if they met the following conditions: (i) conducted as *in vivo* or *in vitro* experiments including both control and ZnO treatment groups; (ii) reported mean values, measures of variability (standard deviation or standard error), and the number of replicates; (iii) reported the experimental duration along with at least one performance parameter or fermentation profile; and (iv) provided clear information on Zn forms, Zn source, and their inclusion levels. After removal of non-original publications, including books; review articles (n = 12); editorials and case reports (n = 3); non-relevant studies (n = 38); and non-English articles (n = 1); thus 28 articles were retained for full-text screening. At the eligibility stage, full-text evaluation was conducted, and studies were excluded for non-relevant parameters (n = 4); the absence of a control group (n = 4); inaccessible full texts (n = 1); and non-ruminant species (n = 2). Consequently, 17 studies met the inclusion criteria and were included in the final meta-analysis, consisting of 5 *in vitro* studies and 12 *in vivo* studies. The study selection process is illustrated in Fig. 1.

### Eligibility criteria

The database comprised a comprehensive set of response variables encompassing both *in vitro* and *in vivo* measurements to capture the multifaceted effects of Zn supplementation on ruminant systems. In the *in vitro* studies, the observed parameters primarily reflected rumen fermentation characteristics, including pH, ammonia concentration, and volatile fatty acid (VFA) profiles such as acetate, propionate, butyrate, isobutyrate, isovalerate, and valerate proportions. Additional indicators, including the acetate-to-propionate ratio and total VFA concentration, were considered to evaluate shifts in fermentation pathways. Digestibility-related variables, such as dry matter digestibility (DMD), organic matter digestibility (OMD), and metabolizable energy (ME), were also included, together with methane production as an indicator of enteric emissions.

In the *in vivo* studies, the parameters were more extensive and categorized into several physiological domains. Rumen fermentability and degradability were assessed through pH, ammonia, VFA composition, total VFA, and dry matter digestibility. Animal performance responses were evaluated using average daily gain (ADG), expressed on both body weight and metabolic body weight bases, as well as dry matter intake (DMI) under similar scaling approaches. Hematological indices, including neutrophil, lymphocyte, monocyte, and eosinophil proportions, were incorporated to reflect immune status. Serum biochemical parameters, such as aspartate aminotransferase (AST), alanine aminotransferase (ALT), and alkaline phosphatase (ALP), were used to indicate metabolic and liver function status. Furthermore, the serum mineral profile, including calcium, phosphate, zinc, and copper concentrations, was evaluated to assess mineral metabolism.

In addition to these primary variables, several other parameters were initially tabulated during database development. However, variables with an insufficient number of comparisons (n < 5) were excluded from the subsequent meta-analysis to ensure statistical robustness and reliability of the effect size.

### Data extraction

For each study, information on publication details, Zn supplementation level and source, feed source, animals, length of experiment, and general information of the studies were extracted (Table 1). Mean values and corresponding measures of dispersion (Standard deviation and standard error) were recorded for each treatment and control group. When variability was reported as standard error (SE), standard deviation (SD) was derived by multiplying the SE by the square root of the sample size (n). Data reported in different units were standardized to common units prior to analysis to ensure consistency across studies. These included standardizing VFA (acetate, propionate, buryrate, isobutyrate, valerate, isovalerate) to mol/100 mol, total VFA to mM, methane to %, and ADG and DMI to g/d BW^0.75 and k^g/d BW^0.75^, respectively.

**Table 1:** Included studies about the utilization of zinc oxide on ration.

No	Reference	level (%)	Form of Zn	ZnO Source	Manufacturer	Feed	Animals	Method of Experiment	length (day) or incubation time (hours)
1	Leško et al., 2025	0 – 0.012*	nZnO	Commercial	SkySpring Nanomaterials. Inc.	Ground barley, wheat bran, soybean meal, limestone	sheep	*in vivo*	107 days
2	Yusuf et al., 2025	0 – 0.06	nZnO	Commercial	nm	Maize bran, wheat offal, palm kernel cake, bone meal, salt	sheep	*in vivo*	85 days
3	Petrič et al., 2024	0 – 0.008*	ZnO and nZnO	Commercial	Sigma-Aldrich	Ground barley, meadow hay	sheep	*in vitro* and *in vivo*	24h and 70 days
4	Xie et al., 2024	0 – 0.003	nZnO	Commercial	Shanghai Macklin Biochemical Co. Ltd	Alfalfa hay, corn, soybean meal, DDGS, corn germ meal, NaCl, limestone, CaHPO₄, vitamin mineral premix	goat	*in vivo*	30 days
5	Youssef et al., 2023	0 – 0.018	nZnO	Laboratory synthesized	-	Corn grain, berseem hay, wheat bran, cottonseed meal, soybean meal	cattle	*in vitro*	24h
6	Yusuf et al., 2023	0 – 0.06	nZnO	Commercial	nm	Maize bran, wheat offal, palm kernel cake, bone meal, salt	goat	*in vivo*	84 days
7	Zhang et al., 2022	0 – 0.008	nZnO	Laboratory synthesized	-	Corn grain, wheat bran, rapeseed meal, DDGS, rice straw, limestone, salt, sodium bicarbonate, vitamin mineral premix	cattle	*in vivo*	60 days
8	Alijani et al., 2020	0 – 0.003*	ZnO and nZnO	Commercial	Kanidam Co and US Research Nano-mate- rials. Inc.	Lucerne hay, barley, maize grains, sodium bicarbonate, NaCl, vitamin mineral premix	sheep	*in vivo*	40 days
9	Hosseini-Vardanjani et al., 2020	0 – 0.003*	ZnO and nZnO	Commercial	Kanidam Co and US Research Nano-mate- rials. Inc.	Lecerne hay, Wheat straw, maize silage, barley grain, maize grain, soybean meal, ammonium phosphate, ammonium sulphate, mineral vitamin premix	cattle	*in vivo*	80 days
10	Fellner et al., 2021	0 – 0.012*	ZnO and nZnO	Commercial	Sigma-Aldrich	Corn silage, whole cottonseed, corn gluten feed, soy hulls, concentrate mix, ground corn	cattle	*in vitro*	24h
11	Abdollahi et al., 2019	0 – 0.005*	ZnO and nZnO	Commercial	Kanidam Co and US Research Nano-mate- rials. Inc.	Alfalfa, barley grain, maize grain, wheat bran, soybean meal, corn gluten meal, sugar beet molasses, fat, sodium bicarbonate, dicalcium phospate, calcium carbonate, toxin binder, sodium chloride, vitamin mineral premix	dairy	*in vivo*	93 days
12	Riazi et al., 2019	0 – 0.006*	ZnO and nZnO	Commercial	US Research Nanomaterials. Inc.	Alfalfa, soybean meal, barley grain, corn grain, salt, mineral premix, and vitamin premix	sheep	*in vitro*	24 and 120h
13	Rodríguez-Maya et al., 2019	0 – 0.006	ZnO	Commercial	Azinsa Óxidos	Corn grain ground, soybean meal, cron stover, canola meal, wheat bran, mineral dan vitamin premix, sodium bicarbonate, calcium carbonate	sheep	*in vivo*	93 days
14	Sarker et al., 2018	0 – 0.1	nZnO	Commercial	US Research Nanomaterials. Inc.	Alfalfa and maize silage	cattle	*in vitro*	72h
15	Puchala et al., 1999	0 – 0.015	ZnO	Commercial	nm	Cottonseed hulls, ground corn, soybean meal, trace mineral salt, calcium carbonate, vitamin premix, dicalcium phospate	goat	*in vivo*	120 days
16	Neathery et al., 1973	0 – 0.003*	ZnO	Commercial	nm	Total mixed ration	dairy	*in vivo*	42 days
17	Miller et al., 1971	0 – 0.06*	ZnO	Commercial	nm	Ground corn grain, soybean oil meal, cottonseed hulls, dicalcium phosphate, marble dust, mineral and vitamin	dairy	*in vivo*	21 days

Nm = not mentioned; * = values are expressed as elemental zinc (Zn) equivalent derived from ZnO supplementation levels.

### Effect size calculation

The magnitude of the treatment effect between ZnO-supplemented and unsupplemented groups was quantified using Hedges'*d* as the standardized effect size metric (Sanchez-Meca & Marin-Martinez, 2010). Hedges’*d* was used because it provides a bias-corrected standardized mean difference that is more appropriate for studies with small or unequal sample sizes. This correction reduces small-sample bias and ensures more robust and comparable effect size across heterogeneous studies (Buck et al., 2022). In this analysis, the unsupplemented group was designated as the control (C) and the ZnO-supplemented group as the experimental (E). A positive effect size indicates a higher observed value in the Zn-supplemented group, whereas a negative value indicates a greater response in the unsupplemented control. The effect size (*d*) was calculated using the following equation:

$$d=\frac{{\bar{X}}^{E}-{\bar{X}}^{C}}{S}\;J$$

Where d = Hedges’*d* effect size; ${\bar{X}}^{E}$ = mean of theexperimental (treatment)group; ${\overset{ˉ}{X}}^{C}$= mean of thecontrolgroup; and S=pooled standard deviation**,** which standardizes the mean difference by the within-study variability. The effect size was computed from the means and pooled standard deviation of each treatment pair, with a small-sample correction factor (*J*) applied to reduce bias associated with limited within-study observations. The pooled standard deviation (*S*) was derived from the sample sizes and standard deviations of both the experimental and control groups. Then, standardized mean difference (SMD) was computed using random-effect model approach implemented in m*etafor* package in R software. To account for non-independent treatments that shared a common control group, a multilevel random-effects meta-analytic model using the “rma.mv” function was used, in which effect sizes were nested within studies (Irawan et al., 2026).

### Heterogeneity assessment

Meta-analysis were conducted when at least three independent comparisons were available for a given outcome. Pooled effect sizes and 95% confidence intervals (CI) were estimated using random-effects models to account for between-study heterogeneity using the DerSimonian and Laird (1986) method. Between-study variance (τ²) was estimated by restricted maximum likelihood (REML). Analyses were performed in JASP software (JASP Team; version 0.98.0). When multiple Zn doses or different zinc forms were reported within the same study, each treatment comparison was retained as a separate effect size because they represented biologically distinct interventions. Consequently, studies contributing multiple comparisons shared a common control group in some cases. To account for the potential non-independence among comparisons originating from the same publication and to minimize pseudo-replication bias, study identity was included as a random effect in the meta-analysis model. Statistical heterogeneity was quantified using Cochran’s Q test (P < 0.10 taken as evidence of heterogeneity) and the I² statistic, which describes the percentage of total variability in effect size due to heterogeneity rather than sampling error. The I² value was calculated using the following formula:

$$I^{2}=100\%\;\times \frac{Q-\left(k-1\right)}{Q}$$

where Q represents Cochran’s heterogeneity statistic, and df corresponds to the degrees of freedom (k − 1, where k is the number of included studies. I² values of approximately 25%, 50%, and 75% were interpreted as low, moderate, and high heterogeneity, respectively (Higgins et al., 2019).

Pre-specified subgroup analysis were used to explore potential effect modifiers, including form of ZnO and ruminant species (cattle, sheep, and goats). These subgroup categories were selected based on biological relevance and expected differences in digestive physiology, rumen function, and nutrient utilization among animal types and experimental conditions. Exploration of heterogeneity through pre-specified subgroup analyses is consistent with current best practices for evidence synthesis and has been widely adopted in recent systematic reviews and meta-analyses (Higgins et al., 2019; Lou et al., 2025).

### Risk of bias assessment

**Fig. 2:**
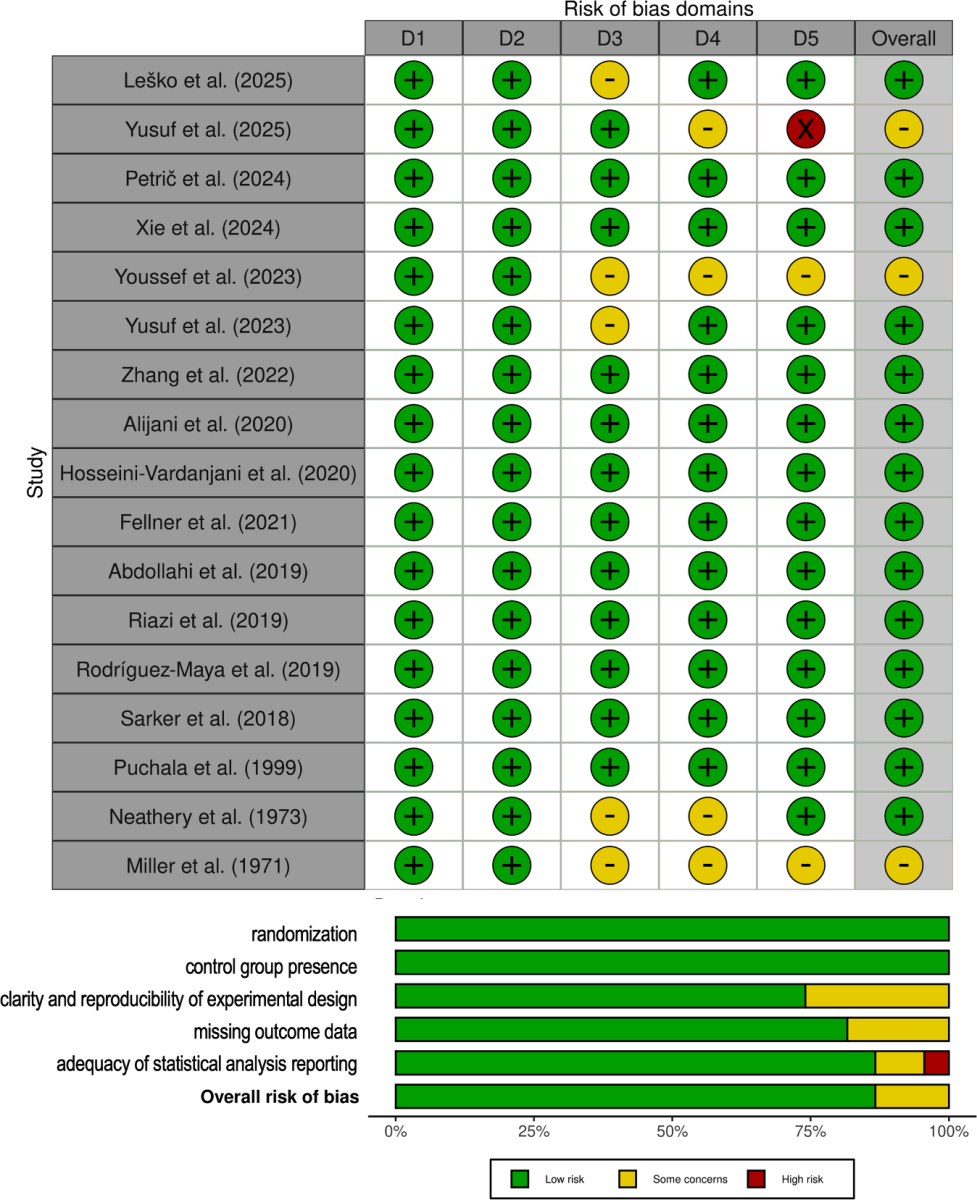
The assessmnet of overall risk of bias (RoB) from the 17 included studies.

A total of 17 selected studies were assessed for methodological quality using risk of bias (RoB) tool encompassing five domains: bias associated with the randomisation process (D1); deviations from intended interventions (D2); incomplete outcome data (D3); outcome measurement bias (D4); and selective reporting of outcomes (D5). For each domain, studies were categorized as low risk, some concerns, or high risk (Sterne et al., 2019). Studies with a high overall risk of bias were excluded from the meta-analysis (Fig. 2).

Publication bias was assessed following Rahmadani et al., 2024 for the primary outcomes of both *in vitro* and *in vivo* studies. This evaluation combined visual inspection offunnel plot asymmetry in combination with a statistical test. Specifically, Egger’s regression test was applied to detect potential small-study effects and asymmetry in the distribution of effect sizes. The combination of graphical assessment and statistical testing is consistent with current recommendations for rigorous evidence synthesis and has been adopted in recent biomedical meta-analyses (Lou et al., 2025). All analyses were performed using JASP (version available at https://jasp-stats.org/). A symmetrical funnel plot, together with a non-significant Egger’s test (P > 0.05), was interpreted as an absence of substantial publication bias, whereas significant asymmetry indicated the potential presence of bias. Furthermore, the trim-and-fill procedure was employed to enhance the evaluation by estimating the number and potential impact of missing studies in the dataset (Shi & Lin, 2019), specifically for the *in vitro* methane production parameter. This approach is similar to the study used in clinical meta-analyses (Lou et al., 2024).

## Results

### Study characteristics

A total of 17 studies published between 1971 and 2025 met the inclusion criteria, comprising both *in vitro* and *in vivo* experiments evaluating zinc supplementation in ruminants. The dataset included multiple zinc forms, *i.e.*, ZnO and nZnO administered across a range of dietary systems and experimental conditions (Table 1). The included studies covered a range of ruminant species. Most were conducted in cattle (47.1%, n = 8), followed by sheep (35.3%, n = 6) and goats (17.6%, n = 3). Most studies evaluated ZnO or nZnO as commercially sourced materials (*e.g.*, Sigma-Aldrich, US Research Nanomaterials Inc., SkySpring Nanomaterials Inc., and other commercial suppliers), while a limited number employed laboratory-synthesized nanoparticles.

Dietary backgrounds were highly heterogeneous, typically based on forage-concentrate mixed rations, including common feed ingredients such as corn grain, barley, soybean meal, wheat bran, alfalfa, silage, and various agro-industrial by-products (*e.g.*, DDGS, cottonseed meal, palm kernel cake, and corn gluten feed). Mineral and vitamin premixes were frequently included to ensure nutritional adequacy.

Across studies, incubation or feeding durations ranged from short-term *in vitro* fermentation (24–120 h) to medium- and long-term *in vivo* trials (21–120 days), reflecting substantial variability in experimental objectives and outcome measurements. Overall, the included studies represent a broad spectrum of experimental conditions, allowing assessment of zinc supplementation effects across different ruminant species, zinc sources, and production systems.

### Descriptive synthesis

Summary statistics for fermentation, digestibility, performance, and physiological variables are presented in Table 2. Overall, ZnO supplementation exerted differential effects between *in vitro* and *in vivo* systems. *in vitro* systems, ZnO supplementation reduced methane production and shifted fermentation toward a more glucogenic profile, as reflected by increased propionate and reduced acetate-to-propionate ratio. Total VFA concentration decreased numerically, whereas digestibility and metabolizable energy showed modest improvements.

**Table 2:** Descriptive statistics of *in vitro* and *in vivo* fermentation parameters in control and treatment groups.

Variable	Unit	NC	Mean		Min		Max		SD	
			Control	Treatment	Control	Treatment	Control	Treatment	Control	Treatment
***In vitro***										
pH		19	6.46	6.52	5.90	6.10	7.19	7.19	0.38	0.38
Ammonia	mg/dL	15	21.15	20.74	13.15	9.02	113.00	113.00	25.52	25.62
Acetate	mol/100 mol	13	60.33	61.11	51.60	49.50	68.60	69.20	5.43	5.52
Propionate	mol/100 mol	12	24.09	24.83	22.75	22.83	26.36	26.50	1.70	0.94
Isobutyrate	mol/100 mol	9	1.51	1.63	0.88	0.60	1.82	2.28	0.46	0.70
Butyrate	mol/100 mol	9	12.42	12.57	11.11	10.91	16.30	16.60	2.25	2.09
Isovalerate	mol/100 mol	9	1.16	0.94	0.14	0.13	4.00	3.50	1.68	1.27
Valerate	mol/100 mol	9	1.05	1.35	0.18	0.18	3.60	5.40	1.48	2.05
Acetate:propionate		9	2.86	2.58	2.20	1.90	4.47	4.58	0.66	0.77
Total volatile fatty acid	mM	13	94.58	86.82	33.50	32.10	193.00	174.00	68.62	52.02
Dry matter digestibility	%	9	48.02	49.37	41.00	43.80	50.84	54.60	4.40	3.57
Organic matter digestibility	%	12	60.48	60.99	56.16	50.64	64.80	67.80	4.51	6.63
Metabolizable energy	MJ/kg DM	6	9.62	9.96	9.62	9.86	9.62	10.08	0.01	0.08
Methane	%	10	14.76	11.70	12.68	6.74	16.15	14.80	1.79	2.51
***In vivo***										
**Fermentability and degradability**										
pH		13	6.36	6.49	5.70	5.66	6.85	6.93	0.46	0.40
Ammonia	mg/dL	11	14.56	11.79	7.01	2.74	20.90	18.00	5.87	5.61
Acetate	mol/100 mol	11	61.50	61.67	49.62	51.36	72.30	70.40	8.08	7.34
Propionate	mol/100 mol	11	23.33	23.91	13.30	14.80	37.53	37.03	8.23	7.46
Isobutyrate	mol/100 mol	11	0.91	0.86	0.47	0.31	1.68	1.74	0.51	0.52
Butyrate	mol/100 mol	11	12.72	11.88	10.25	9.01	16.83	17.08	2.34	2.70
Isovalerate	mol/100 mol	11	0.54	0.61	0.06	0.04	1.63	1.92	0.49	0.58
Valerate	mol/100 mol	11	1.07	1.08	0.40	0.42	1.95	2.65	0.46	0.61
Acetate:propionate		7	3.42	3.28	1.32	1.39	5.46	4.77	1.54	1.33
Total volatile fatty acid	mM	11	63.44	65.20	36.60	36.00	92.60	104.00	16.79	22.14
Dry matter digestibility	%	6	66.90	69.98	60.30	61.30	70.40	75.50	5.12	5.89
**Performance**										
Average daily gain	g/d	8	420.86	490.56	55.90	50.50	950.00	1170.00	344.64	415.18
Average daily gain	g/d BW^0.75^	8	22.48	25.53	5.16	4.57	44.04	51.55	15.12	17.22
Dry matter intake	g/d	8	1438.63	1498.04	145.00	138.33	1766.00	1968.00	536.35	580.68
Dry matter intake	g/d BW^0.75^	8	91.53	93.88	1.59	1.51	173.26	165.29	46.69	44.70
**Hematological parameters**										
Neutrophil	%	6	32.27	30.93	27.50	26.33	39.65	35.87	5.80	3.67
Lymphocyte	%	6	63.82	65.06	56.03	59.24	68.75	71.00	6.10	4.52
Monocyte	%	6	1.64	1.45	1.25	0.67	2.00	2.14	0.34	0.60
Eosinophil	%	6	1.66	1.66	1.00	1.00	2.32	2.77	0.59	0.84
**Serum biochemical parameters**										
Aspartate aminotransferase	U/L	8	82.67	86.35	53.60	61.30	111.00	125.00	22.68	26.40
Alanine aminotransferase	U/L	8	32.20	33.61	16.00	15.33	40.50	40.70	10.18	10.61
Alkaline phosphatase	U/L	6	133.40	157.86	70.20	66.70	165.00	207.00	48.95	65.92
**Serum mineral profile**										
Calcium	mg/dL	6	11.78	11.67	8.59	8.45	15.00	15.23	2.87	3.01
Phospate	mg/dL	6	7.27	8.01	5.53	5.43	9.05	9.90	1.57	1.96
Zinc	µg/dL	8	119.36	126.48	82.10	87.83	171.33	185.20	34.70	32.41
Copper	µg/dL	8	103.34	106.38	94.60	90.60	112.00	141.47	6.66	15.42

Under *in vivo* conditions, ZnO supplementation generally improved fermentation efficiency and animal productivity. Total VFA concentration and improved performance indicators, including ADG and DMI. Circulating Zn concentration increased, indicating enhanced mineral bioavailability. The relatively proportional increased in intake and growth suggests improved productive efficiency rather than disproportionate feed consumption.

Physiologically, ZnO supplementation increased circulating zinc concentration (119.36 vs. 126.48 µg/dL in control and treatment groups, respectively), confirming enhanced mineral bioavailability. Hematological parameters, including leukocyte subpopulations, remained largely stable across treatments, with neutrophils (32.27 vs. 30.93%), lymphocytes (63.82 vs. 65.06%), monocytes (1.64 vs. 1.45%), and eosinophils (1.66 vs. 1.66%) showing minimal variation between control and ZnO-supplemented groups. Similarly, serum biochemical markers of liver function showed no clear adverse alterations, with aspartate aminotransferase (82.67 vs. 86.35 U/L), alanine aminotransferase (32.20 vs. 33.61 U/L), and alkaline phosphatase (133.40 vs. 157.86 U/L) indicating maintained hepatic integrity. Mineral status also remained relatively stable, with calcium (11.78 vs. 11.67 mg/dL), phosphate (7.27 vs. 8.01 mg/dL), and copper (103.34 vs. 106.38 µg/dL) showing only minor variations between control and treatment groups, although a modest increase in circulating zinc was observed.

### Meta-analysis of zinc supplementation on *in vitro* fermentation

The pooled effects of ZnO supplementation on *in vitro* fermentation are summarized in Table 3. ZnO exerted a significant antimethanogenic effect (SMD = −1.281; P < 0.001) with no detectable heterogeneity (I² = 0%). This response was accompanied by a significant increase in propionate concentration (SMD = 0.521; P = 0.026) and a marked reduction in the acetate-to-propionate ratio (SMD = −1.226; P < 0.001).

**Table 3:** Cumulative meta-analysis of the effects of zinc oxide on *in vitro* fermentation parameters.

Variable	Unit	NC	SMD	Lower	Upper	Std. error	*P*-Value	τ^2^	Q	Q. *P*-value	I^2^
pH		19	−0.474	−1.419	0.471	0.482	0.326	3.026	82.343	<0.001	78.140
Ammonia	mg/dL	15	−0.287	−0.952	0.378	0.339	0.398	0.913	31.797	0.004	55.971
Acetate	mol/100 mol	13	−0.015	−0.476	0.446	0.235	0.949	0.125	14.569	0.266	17.631
Propionate	mol/100 mol	12	0.521	0.062	0.98	0.234	0.026	0.000	9.034	0.619	0.000
Isobutyrate	mol/100 mol	9	0.305	−0.189	0.8	0.252	0.226	0.000	4.936	0.764	0.000
Butyrate	mol/100 mol	9	0.024	−0.463	0.511	0.248	0.923	0.000	2.222	0.973	0.000
Isovalerate	mol/100 mol	9	0.239	−0.42	0.898	0.336	0.478	0.351	12.408	0.134	35.526
Valerate	mol/100 mol	9	0.262	−0.242	0.767	0.258	0.308	0.008	8.105	0.423	1.292
Acetate:propionate		9	−1.226	−1.952	−0.5	0.37	<0.001	0.459	13.07	0.109	38.792
Total volatile fatty acid	mM	13	0.222	−0.268	0.711	0.25	0.375	0.204	16.135	0.185	25.627
Dry matter digestibility	%	9	0.028	−1.348	1.404	0.702	0.968	3.483	41.363	<0.001	80.659
Organic matter digestibility	%	12	1.058	−0.337	2.453	0.712	0.137	4.738	54.499	<0.001	79.816
Metabolizable energy	MJ/kg DM	6	2.034	1.223	2.845	0.414	<0.001	0.000	1.074	0.956	0.000
Methane	%	10	−1.281	−1.818	−0.743	0.274	<0.001	0.000	7.711	0.564	0.000

NC = number of comparison; SMD = standardized mean difference; τ² **=** estimated between-study variance (heterogeneity); Q **=** Cochran's heterogeneity statistic;Q P-value **=**P-value associated with Cochran's Q test for heterogeneity;I² = percentage of between-study heterogeneity rather than sampling error.

Metabolizable energy was significantly elevated (SMD = 2.034; P < 0.001), whereas no significant effects were detected for pH, ammonia, total VFA, or digestibility variables (P > 0.05). Substantial between-study heterogeneity was observed for several parameters, including pH (I² = 78.14%) and DMD (I² = 80.66%).

### Meta-analysis of zinc oxide supplementation on *in vivo* responses

The pooled *in vivo* responses are presented in Table 4. ZnO supplementation significantly reduced ruminal ammonia concentration (SMD = −0.869; P < 0.001). Total VFA concentration was modestly increased (SMD = 0.520; P = 0.018), although individual VFA proportions and ruminal pH were not significantly affected.

**Table 4:** Cumulative meta-analysis of the effects of zinc oxide on *in vivo* fermentation and performance parameters.

Variable	Unit	NC	SMD	Lower	Upper	Std. error	*P*-Value	τ^2^	Q	Q *P*-value	I^2^
**Fermentability and degradability**											
pH		13	0.183	−0.085	0.451	0.137	0.18	0.000	10.462	0.575	0.000
Ammonia	mg/dL	11	−0.869	−1.333	−0.404	0.237	<0.001	0.354	23.622	0.009	57.666
Acetate	mol/100 mol	11	0.046	−0.235	0.327	0.143	0.749	0.000	3.017	0.981	0.000
Propionate	mol/100 mol	11	0.117	−0.165	0.398	0.144	0.417	0.000	4.104	0.943	0.000
Isobutyrate	mol/100 mol	11	−0.113	−0.394	0.168	0.143	0.431	0.000	1.833	0.997	0.000
Butyrate	mol/100 mol	11	−0.263	−0.546	0.019	0.144	0.068	0.000	3.574	0.965	0.000
Isovalerate	mol/100 mol	11	−0.002	−0.283	0.28	0.144	0.99	0.000	4.554	0.919	0.000
Valerate	mol/100 mol	11	−0.028	−0.31	0.253	0.144	0.843	0.000	5.253	0.874	0.000
Acetate:propionate		7	−0.01	−0.369	0.349	0.183	0.956	0.000	1.757	0.941	0.000
Total volatile fatty acid	mM	11	0.52	0.09	0.949	0.219	0.018	0.281	21.422	0.018	53.318
Dry matter digestibility	%	6	3.669	1.797	5.541	0.955	<0.001	4.378	58.710	<0.001	91.484
**Performance**											
Average daily gain	g/d	8	0.565	0.185	0.945	0.194	0.004	0.074	9.297	0.232	24.705
Average daily gain	g/d BW^0.75^	8	0.545	0.144	0.945	0.204	0.008	0.108	10.335	0.17	32.270
Dry matter intake	kg/d	8	0.649	0.1	1.197	0.28	0.02	0.395	19.094	0.008	63.339
Dry matter intake	kg/d BW^0.75^	8	0.642	0.081	1.203	0.286	0.025	0.425	19.984	0.006	64.973
**Hematological parameters**											
Neutrophil	%	6	−0.412	−0.989	0.165	0.294	0.162	0.216	8.62	0.125	41.995
Lymphocyte	%	6	0.48	−0.205	1.165	0.35	0.17	0.412	11.735	0.039	57.394
Monocyte	%	6	−0.485	−1.226	0.256	0.378	0.199	0.531	13.611	0.018	63.264
Eosinophil	%	6	−0.317	−1.014	0.381	0.356	0.373	0.444	12.348	0.03	59.507
**Serum biochemical parameters**											
Aspartate aminotransferase	U/L	8	−0.201	−0.806	0.404	0.309	0.515	0.460	18.374	0.01	61.903
Alanine aminotransferase	U/L	8	0.214	−0.292	0.719	0.258	0.408	0.245	13.175	0.068	46.868
Alkaline phosphatase	U/L	6	0.822	0.068	1.576	0.385	0.033	0.586	15.277	0.009	67.270
**Serum mineral profile**											
Calcium	mg/dL	6	0.144	−0.354	0.642	0.254	0.571	0.140	7.876	0.163	36.516
Phospate	mg/dL	6	0.67	−0.14	1.481	0.413	0.105	0.718	18.466	0.002	72.924
Zinc	µg/dL	8	0.704	−0.028	1.437	0.374	0.06	0.817	27.433	<0.001	74.483
Copper	µg/dL	8	0.108	−0.395	0.612	0.257	0.673	0.272	14.784	0.039	52.650

NC = number of comparison; SMD = standardized mean difference; τ² **=** estimated between-study variance (heterogeneity); Q **=** Cochran's heterogeneity statistic;Q P-value **=**P-value associated with Cochran's Q test for heterogeneity;I² = percentage of between-study heterogeneity rather than sampling error.

Performance outcomes showed consistent positive responses. ADG increased significantly (SMD = 0.565; P = 0.004), as did DMI (SMD = 0.649; P = 0.020). When expressed relative to metabolic body weight, these responses remained significant (P < 0.05). DMD was significantly increased (SMD = 3.669; P < 0.001), with substantial heterogeneity among studies (I² = 96.04%).

Regarding physiological responses, ALP activity increased significantly (SMD = 0.822; P = 0.033), whereas other serum biochemical indicators remained unaffected. Serum Zn concentration showed a near-significant increase (SMD = 0.704; P = 0.060), indicating a consistent directional response despite variability among studies.

### Subgroup analysis by zinc oxide form and animal species

**Fig. 3:**
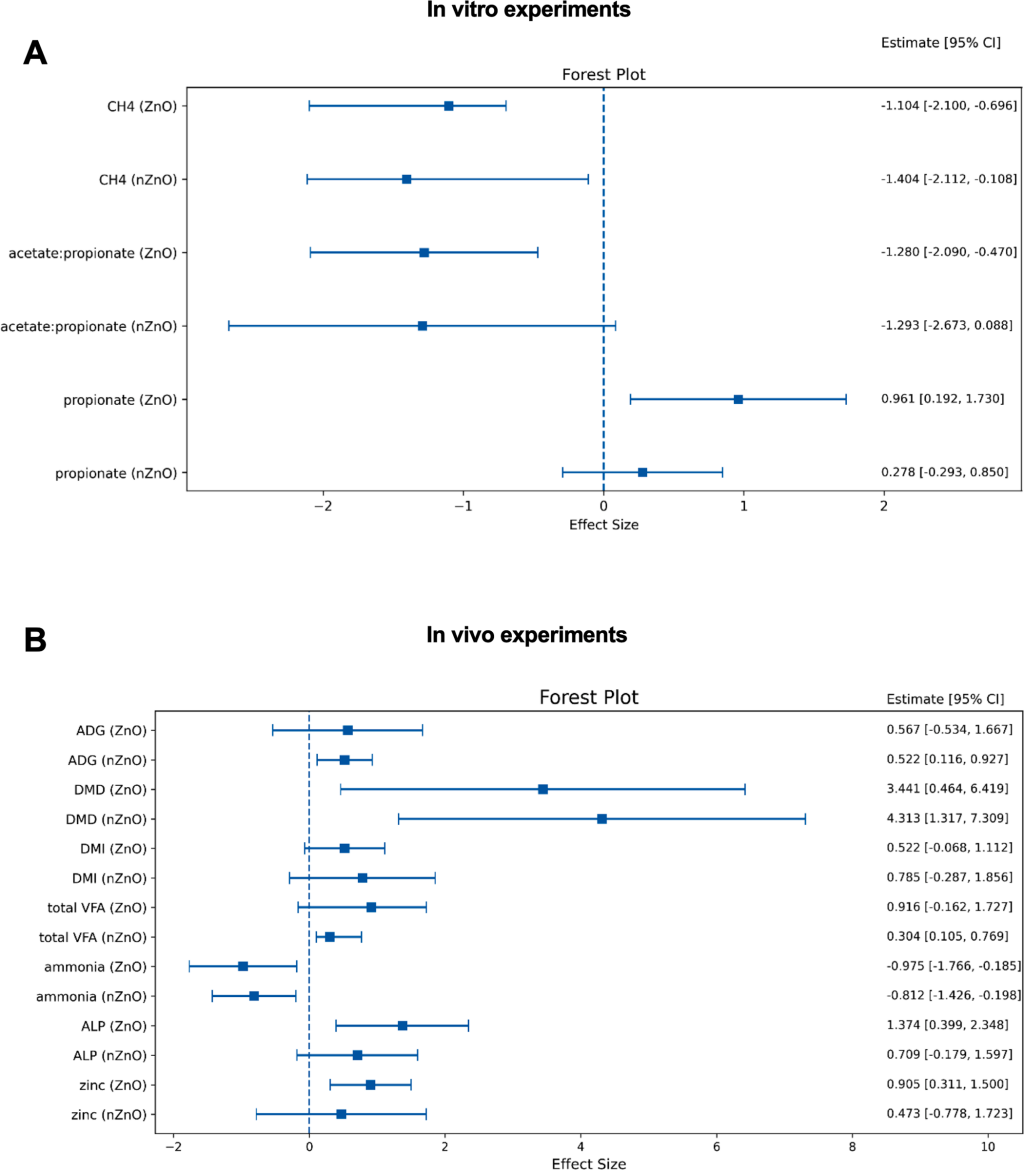
Forest plot of subgroup meta-analysis comparing the effects of zinc oxide (ZnO) and nano-zinc oxide (nZnO) supplementation on *in vitro* parameters (A) and *in vivo* performance, rumen fermentation, and metabolic parameters (B). Effect sizes are presented as standardized mean differences with 95% confidence intervals (CI). The vertical dashed line represents the null effect (no difference between treatments). Positive values indicate a greater response in the treatment group compared with the control, whereas negative values indicate a lower response. Subgroup analysis includes average daily gain (ADG), dry matter digestibility (DMD), dry matter intake (DMI), total volatile fatty acids (total VFA), ammonia concentration, alkaline phosphatase (ALP), and zinc concentration.

Subgroup analysis revealed differential responses depending on Zn form (Fig. 3). *in vitro* systems, both ZnO and nZnO significantly reduced methane production, with nZnO showing a numerically greater antimethanogenic effect (SMD = −1.404; P < 0.001) than conventional ZnO (SMD = −1.104; P < 0.001). ZnO supplementation also significantly decreased the acetate-to-propionate ratio (SMD = −1.280; P < 0.001) and increased propionate concentration (SMD = 0.961; P < 0.001), whereas the effects of nZnO on the acetate-to-propionate ratio and propionate production were not significant (P > 0.05).

*in vivo* studies, both ZnO and nZnO improved several productive and fermentative parameters, although response magnitude differed between zinc sources. nZnO produced significant increased in average daily gain (SMD = 0.522; P = 0.014), DMD (SMD = 4.313; P = 0.01), and total VFA concentration (SMD = 0.304; P = 0.011), while simultaneously reducing ruminal ammonia concentration (SMD = −0.812; P < 0.001). Conventional ZnO also improved DMD (SMD = 3.441; P < 0.001) and decreased ammonia concentration (SMD = −0.975; P < 0.001), and enhanced serum Zn concentration (SMD = 0.905; P = 0.018) and ALP activity (SMD = 1.374; P < 0.001), although its effects on growth performance and intake were less consistent. Overall, nZnO appeared to exert stronger effects on digestibility and growth-related variables, whereas conventional ZnO showed more pronounced responses in mineral status and biochemical indicators.

The present meta-analysis also demonstrated that dietary ZnO supplementation in ruminants improved ruminal fermentation, nutrient utilization, and animal performance across ruminant species while maintaining physiological stability (Table 5). Although subgroup analysis indicated variations in effect size among ruminant species, no formal statistical test for differences between subgroups (cattle *vs* sheep *vs* goat) was conducted. Therefore, comparisons across species should be interpreted descriptively rather than as statistically significant differences in effect magnitude. Ruminal pH and individual VFA proportions were unaffected in cattle and sheep (P > 0.05), indicating preservation of rumen fermentation balance. However, ammonia concentration decreased significantly in cattle (SMD = −1.256; P < 0.001) and sheep (SMD = −0.779; P = 0.005), accompanied by increased total VFA concentration in cattle (SMD = 0.660; P = 0.042) and a tendency toward higher VFA production in sheep (P = 0.073). DMD was improved significantly in both cattle (SMD = 1.713%; P < 0.001) and sheep (SMD = 5.716%; P = 0.002). Consistently, ADG increased significantly in cattle and sheep (P < 0.05), whereas DMI remained unchanged, suggesting enhanced feed efficiency rather than increased feed consumption. Hematological responses were generally stable, although goats exhibited reduced monocyte (SMD = −1.926%; P < 0.001) and eosinophil percentages (SMD = −1.671%; P < 0.001).

**Table 5:** Species-based subgroup meta-analysis of zinc oxide effects on *in vivo* fermentation and performance parameters.

Variable	SMD	Lower	Upper	Std. error	*P*-Value
**Fermentability and degradability**					
pH					
Cattle	0.05	−0.571	0.671	0.317	0.874
Sheep	0.214	−0.084	0.511	0.152	0.159
Ammonia, mg/dL					
Cattle	−1.256	−1.938	−0.575	0.348	<0.001
Sheep	−0.779	−1.325	−0.234	0.278	0.005
Acetate, mol/100 mol					
Cattle	0.401	−0.225	1.027	0.319	0.209
Sheep	−0.044	−0.359	0.271	0.161	0.784
Propionate, mol/100 mol					
Cattle	−0.256	−0.879	0.367	0.318	0.42
Sheep	0.212	−0.103	0.528	0.161	0.188
Isobutyrate, mol/100 mol					
Cattle	−0.106	−0.727	0.514	0.316	0.737
Sheep	−0.114	−0.429	0.201	0.161	0.477
Butyrate, mol/100 mol					
Cattle	−0.289	−0.915	0.336	0.319	0.365
Sheep	−0.136	−0.824	0.552	0.351	0.699
Isovalerate, mol/100 mol					
Cattle	0.079	−0.541	0.7	0.317	0.802
Sheep	−0.023	−0.339	0.293	0.161	0.888
Valerate, mol/100 mol					
Cattle	−0.078	−0.698	0.542	0.316	0.805
Sheep	−0.016	−0.332	0.301	0.161	0.923
Acetate:propionate					
Cattle	0.287	−0.336	0.91	0.318	0.367
Sheep	−0.158	−0.597	0.282	0.224	0.481
Total volatile fatty acid, mM					
Cattle	0.66	0.023	1.298	0.325	0.042
Sheep	0.488	−0.046	1.021	0.272	0.073
Dry matter digestibility, %					
Cattle	1.713	0.77	2.656	0.481	<0.001
Sheep	5.716	2.177	9.256	1.806	0.002
					
**Performance**					
Average daily gain, g/d					
Cattle	0.96	0.422	1.497	0.274	<0.001
Sheep	0.488	0.029	0.947	0.234	0.037
Average daily gain, g/d BW^0.75^					
Cattle	0.956	0.418	1.494	0.274	<0.001
Sheep	0.466	0.007	0.924	0.234	0.047
Dry matter intake, kg/d					
Cattle	0.51	−0.357	1.377	0.442	0.249
Sheep	0.745	−0.039	1.529	0.4	0.063
Dry matter intake, kg/d BW^0.75^					
Cattle	0.51	−0.401	1.421	0.465	0.273
Sheep	0.733	−0.062	1.528	0.406	0.071
					
**Hematological parameters**					
Neutrophil, %					
Sheep	−0.237	−0.734	0.261	0.254	0.351
Goat	−1.057	−2.816	0.703	0.898	0.239
Lymphocyte, %					
Sheep	0.13	−0.365	0.626	0.253	0.606
Goat	1.594	−0.129	3.317	0.879	0.07
Monocyte, %					
Sheep	0.075	−0.415	0.565	0.25	0.765
Goat	−1.926	−2.9	−0.951	0.497	<0.001
Eosinophil, %					
Sheep	0.211	−0.28	0.703	0.251	0.399
Goat	−1.671	−2.6	−0.742	0.474	<0.001
					
**Serum biochemical parameters**					
Aspartate aminotransferase, U/L					
Sheep	0.234	−0.161	0.628	0.201	0.245
Goat	−1.969	−3.792	−0.146	0.93	0.034
Alanine aminotransferase, U/L					
Sheep	0.143	−0.25	0.537	0.201	0.474
Goat	0.828	−2.572	4.228	1.735	0.633
Alkaline phosphatase, U/L					
Sheep	0.901	0.427	1.376	0.242	< 0.001
Goat	0.766	−2.701	4.233	1.769	0.665
					
**Serum mineral profile**					
Calcium, mg/dL					
Cattle	−0.055	−0.675	0.565	0.316	0.862
Sheep	−0.308	−0.932	0.315	0.318	0.332
Goat	1.154	0.287	2.021	0.442	0.009
Phosphate, mg/dL					
Cattle	0.054	−0.566	0.674	0.316	0.864
Sheep	−0.005	−0.625	0.615	0.316	0.988
Goat	2.507	1.301	3.714	0.616	<0.001
Zinc, µg/dL					
Cattle	0.805	0.063	1.547	0.379	0.034
Sheep	1.311	0.812	1.809	0.254	<0.001
Goat	−1.175	−4.533	2.183	1.713	0.493
Copper, µg/dL					
Cattle	0.082	−0.539	0.703	0.317	0.796
Sheep	−0.251	−0.703	0.202	0.231	0.278
Goat	1.513	−1.254	4.279	1.412	0.284

### Meta-regression analysis

Meta-regression analyses from *in vitro* dataset (Supplementary File; Table S1) indicated that most of the evaluated moderators (Zn form, animal species, breed, and type of animal) did not significantly influence the responses of acetate, propionate, total VFA, or ruminal pH (P > 0.05). Meta-regression analysis also identified Zn dose as a significant moderator of ADG responses (P = 0.040; Supplementary Table S2), indicating that the magnitude of the growth response to Zn supplementation varied according to the supplementation level within the evaluated dose range. For DMI, breed was the only significant moderator (P = 0.004), and its inclusion reduced residual heterogeneity (QE = 2.91, P = 0.233; τ² = 0), suggesting that breed accounted for a considerable proportion of between-study variation. In contrast, none of the evaluated moderators significantly affected ruminal pH, acetate, propionate, total VFA, or NH₃ concentrations (P > 0.05). However, these findings should be interpreted with caution because the number of studies available for each outcome was relatively small (N = 8–17), which may have limited the statistical power of the meta-regression analyses and contributed to the low residual heterogeneity (τ²) observed for several models. Therefore, the apparent reduction in heterogeneity may reflect limited data availability rather than a complete explanation of between-study variability.

### Publication bias

**Fig. 4:**
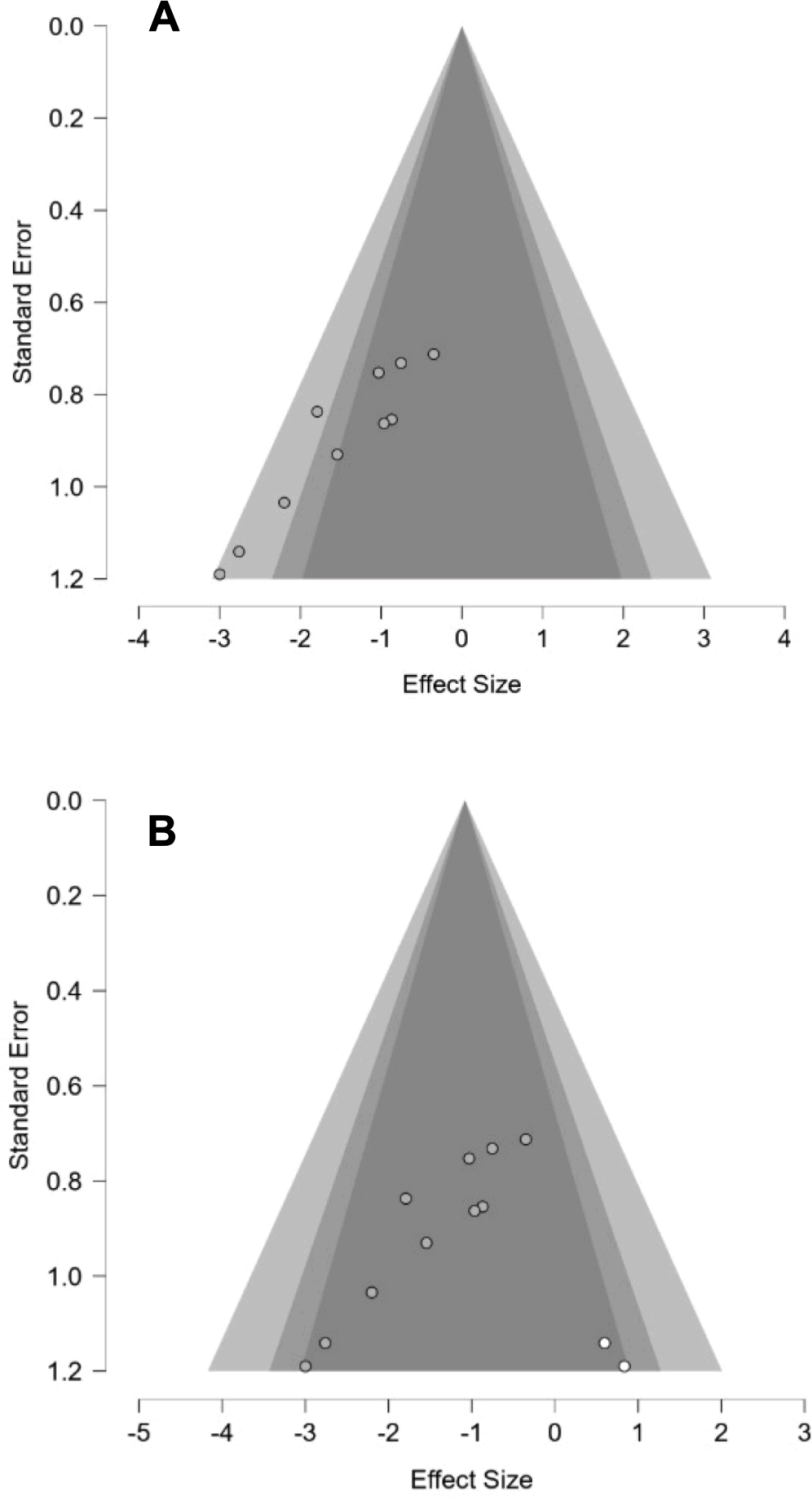
Funnel plot of publication bias on methane *in vitro* fermentation. (A) original plot, (B) after applying the trim-and-fill method. Two potentially missing studies were imputed on the right side of the funnel plot (indicated by white circles), resulting in a more symmetrical distribution of studies around the pooled effect size.

Publication bias for methane production on *in vitro* studies was assessed using visual inspection of funnel plots in conjunction with statistical testing (Fig. 4). The original funnel plot showed noticeable asymmetry, suggesting small-study effects. This observation was confirmed by Egger’s regression test, which indicated significant asymmetry (z = −2.559; P = 0.01), providing statistical evidence of potential publication bias.

To further evaluate the robustness of the observed effect, the trim-and-fill method was applied. This analysis imputed two potentially missing studies, yielding an adjusted SMD of −1.082. The corrected effect size remained statistically significant, with a 95% confidence interval ranging from −1.592 to −0.572. The adjusted SMD was consistent in direction and magnitude with the original pooled effect, indicating that the antimethanogenic effect of Zn supplementation is robust despite moderate publication bias.

**Fig. 5:**
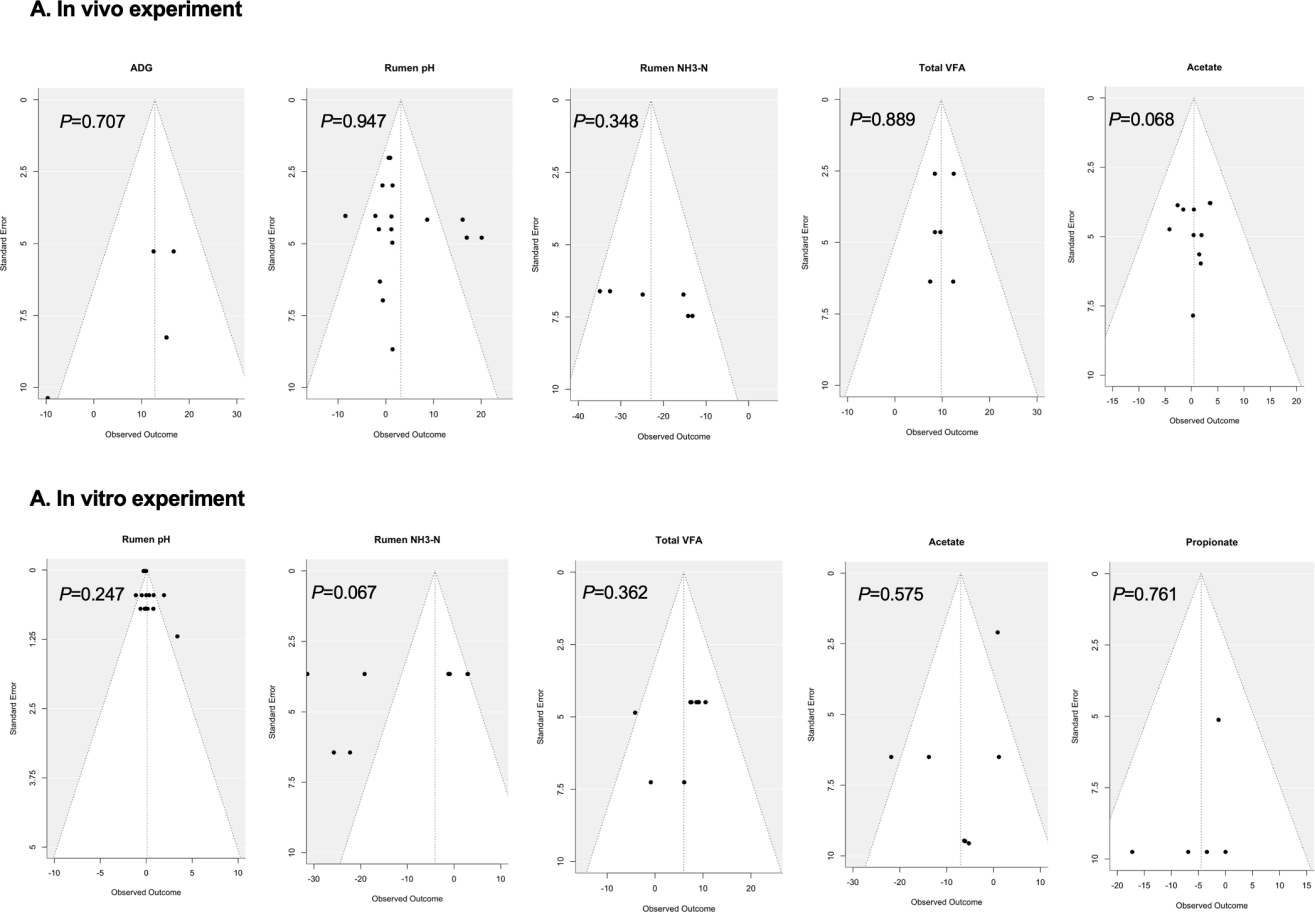
Funnel plots of publication bias on average daily gain (ADG), rumen pH, rumen NH_3—_N, total VFA, acetate, and propionate *in vivo* and *in vitro* studies. No significant funnel plot asymmetry was detected using Egger’s regression test (P > 0.05), suggesting a low of publication bias.

Publication bias was also assessed for other outcomes with sufficient numbers of comparisons using funnel plots and Egger's regression tests (Fig. 5). No significant funnel plot asymmetry was detected for ADG (P = 0.707), rumen pH (P = 0.947), rumen NH_3—_N (P = 0.348), total VFA (P = 0.889), acetate (P = 0.068), *in vitro* rumen pH (P = 0.247), rumen NH_3—_N (P = 0.067), total VFA (P = 0.362), acetate (P = 0.575), or propionate (P = 0.761). These results suggest a low of publication bias for these parameters. Although acetate *in vivo* and rumen NH_3—_N *in vitro* showed values close to the significance threshold, neither reached statistical significance.

## Discussion

### Utilization of zinc oxide on ration

The use of ZnO in livestock rations across various recent studies spanning 1971 to 2025, specifically comparing ZnO and nZnO. Research trends indicate diverse outcomes, with a transition from conventional sources to nanotechnology applications. Trials of ZnO levels in rations used varied within a precise range from 0.003% to 0.06%. This demonstrates different experiments by researchers to identify the optimal dosage that maximizes livestock performance while mitigating environmental pollution and toxicological risks. Regarding the origin of the materials, the majority of the mineral sources were obtained from reputable commercial manufacturers, including Sigma-Aldrich, Kanidam Co., US Research Nanomaterials Inc., SkySpring Nanomaterials, and Azinsa Óxidos. This sourcing ensures the standardization and purity of the substances tested across both *in vitro* and *in vivo* parameters. However, some studies have showcased noteworthy innovations; for instance, researchers such as Youssef et al., 2023 and Zhang et al., 2021 utilized laboratory-synthesized nZnO in their investigations.

The composition of the basal diets across studies reflects a ruminant feeding system that integrates energy, fiber, and high-protein sources. The use of structural fiber materials, such as various types of hay, silage, and rice straw, combined with energy sources such as corn, meal, and other industrial byproduct grains, can influence rumen feed flow rates and create a complex rumen chemical environment both *in vitro* and *in vivo*. The presence of a varied feed mixture is crucial for evaluating the kinetic behavior of ZnO particles in the digestive system (Fellner et al., 2021). Specifically, the interaction between the surface charge of nanoparticles and functional groups in feed fibers (such as cellulose and lignin) can affect their bioavailability to rumen microbes (Kholif et al., 2025). Furthermore, the inclusion of buffer minerals, such as sodium bicarbonate, is vital for maintaining rumen pH stability, thereby preventing the degradation or agglomeration of nanoparticles under acidic conditions.

The synergy between ZnO supplementation and macrominerals, such as calcium phosphate, underscores efforts to optimize mineral homeostasis in livestock. This nutritional balance ensures that the effects of ZnO supplementation are not hampered by mineral antagonism but instead work synchronically to support metalloenzyme synthesis and microbial proteolytic activity (Wang et al., 2017). Furthermore, the bioavailability of dietary Zn is significantly influenced by the presence of other nutritional elements, which may either promote or inhibit its absorption. Specifically, elevated levels of calcium have been shown to antagonize zinc uptake. This inhibitory effect occurs through the formation of insoluble calcium-zinc-phosphate complexes within the intestinal lumen, thereby sequestering zinc and rendering it unavailable for absorption (Khaksar et al., 2025). Thus, selecting comprehensive ration components to more accurately livestock responses, including improving fermentation efficiency.

### Effects of zinc oxide supplementation on *in vitro* fermentation

The results demonstrate that ZnO supplementation alters the VFA profile in the *in vitro* rumen system. The increase in propionate concentration indicates a shift in the fermentation pathway toward a more efficient, energetically favorable direction. This suggests an increase in the primary hydrogen sink within the rumen, which is frequently associated with improved energy utilization efficiency in ruminants (Jo et al., 2024). Findings from Fellner et al., 2021 align with this meta-analysis, reporting that Zn can modulate cellulolytic and amylolytic microbial activities, which in turn, drive fermentation toward higher propionate production. The shift in fermentation patterns in this study is further supported by a decrease in the acetate-to-propionate ratio, a critical indicator of rumen fermentation efficiency. Biochemically, acetate production releases free H_2_, whereas propionate production utilizes it. Therefore, Zn likely acts as a microbial population modulator, suppressing acetate-producing bacteria while favoring propionate-producing bacteria (Arelovich et al., 2000). Furthermore, changes in propionate proportions are due to physiological adjustments to maintain homeostasis (Jo et al., 2024). The source of Zn utilized in this meta-analysis appears to have influenced specific microbial organisms, whereas ZnO increased rumen-soluble Zn and minimized pH fluctuation (Fellner et al., 2021).

One of the most important findings of this meta-analysis was the reduction in methane emissions. This decrease in methane was accompanied by an increase in propionate production. The observed response could be associated with altered microbial fermentation dynamics, including possible inhibition of methanogenic archaea and competitive hydrogen utilization by propionate-producing bacteria. The antibacterial effects of ZnO may be associated with its ability to induce oxidative stress and metabolic disruption in certain microbial populations. Geetha et al., 2020 proposed a mechanism involving the formation of hydrogen peroxide (H₂O₂), a reactive oxygen species that can penetrate microbial cell walls and induce oxidative damage to proteins, lipids, and DNA, thereby impairing normal microbial metabolism. Although Zn is an essential component of numerous metalloenzymes involved in fatty acid biosynthesis and DNA/RNA polymerase activity (Najafzadeh et al., 2013), its antimicrobial effects may be selective. ZnO may suppress certain microbial groups, including methanogens or acetate-producing bacteria, while potentially supporting propionigenic bacteria by supplying Zn as an enzymatic cofactor (Yusof et al., 2019). Therefore, the combination of Zn bioavailability and antimicrobial activity may contribute to the observed improvements in rumen fermentation efficiency without severely impairing overall fermentation activity.

Reducing methane emissions not only has environmental implications through mitigation of greenhouse gas production, but may also improve energy utilization by decreasing gaseous energy losses. Intercellular hydrogen transfer between methanogens and the fermentative microbial community, including protozoa, fungi, and bacteria, plays an important role in regulating ruminal H₂ concentrations, and excessive H₂ accumulation has been reported to inhibit hydrogenase activity and carbohydrate oxidation (Tseten et al., 2022). In the present meta-analysis, the significant increase in metabolizable energy may suggest improved energy availability from feed, potentially associated with shifts in fermentation pathways toward greater propionate production and lower methane formation. Although DMD and OMD showed positive but statistically non-significant SMD, the observed fermentation shifts may still have enhanced the proportion of feed energy retained by the animal. These findings are consistent with Sadegzadeh-Sadat et al. (2021), who reported increased metabolizable energy in zinc-supplemented late-pregnant ewes. In addition, Zn functions as a cofactor for >300 enzymes involved in carbohydrate and energy metabolism (Yatoo et al., 2013), which could partly explain the improvement in energy utilization efficiency despite the absence of significant changes in total digestibility.

In the rumen, Zn is required for several microbial enzymatic processes, including cellulase and protease activity, and may influence fermentation patterns and microbial protein synthesis. However, Zn is relatively insoluble at neutral ruminal pH, and its bioavailability may be influenced by dietary fiber, phytate complexation, and interactions with other minerals, such as *Ca*, Cu, and Fe. These factors may partly explain why some rumen fermentation responses remained modest or inconsistent across studies. In this meta-analysis, the absence of significant effects on ruminal pH and several VFA fractions suggests that Zn supplementation at the evaluated doses did not severely disrupt rumen fermentation stability. Overall, the present findings support the potential application of Zn supplementation to improve energy utilization while contributing to more environmentally sustainable ruminant production systems.

### Effects of zinc oxide supplementation on *in vivo* fermentation and growth performance

ZnO supplementation exhibited variable effects on *in vivo* ruminal fermentation parameters. One of the major findings of the present study was the significant reduction in rumen NH_3_ concentration. This decline in NH_3_ may suggest improved nitrogen utilization efficiency, potentially associated with altered ruminal protein degradation and NH_3_ assimilation by rumen microbes. Ji et al. (2021) reported that hydroxypeptidase is a Zn-dependent enzyme involved in peptide hydrolysis and amino acid release during protein metabolism. In ruminants, Zn²⁺ absorption occurs primarily in the rumen, abomasum, and small intestine, although its availability may vary depending on the complex digestive physiology and dietary interactions present within the gastrointestinal tract (Cai et al., 2025).

ZnO supplementation also demonstrated positive effects on the end products of ruminal microbial fermentation, particularly an increase in total VFA concentration. Given that VFA contributes approximately 70–80% of the metabolizable energy supply in ruminants, the increase in total VFA concentration may indicate enhanced fermentation efficiency under ZnO supplementation. This response may be associated with the role of Zn as a cofactor for fibrolytic and amylolytic enzymes involved in carbohydrate degradation (Arelovich et al., 2008; Yusof et al., 2019). Interestingly, despite the increase in total VFA concentration, the relative proportions of individual VFAs and ruminal pH remained stable, suggesting that Zn supplementation enhanced overall fermentative activity without substantially disrupting primary fermentation pathways or rumen ecosystem stability. Maintenance of ruminal pH within the physiological range is important for sustaining cellulolytic bacterial activity and optimal fiber degradation (Yusof et al., 2019).

The present meta-analysis also demonstrated that ZnO supplementation improved ADG and DMI. These improvements may reflect greater nutrient utilization efficiency and enhanced energy availability associated with increased VFA production (Min et al., 2019). Previous studies have suggested that adequate Zn supplementation may improve productivity through multiple physiological pathways, including optimizing digestive efficiency, nutrient absorption, intestinal barrier integrity, and maintaining gut microbial balance (Zarghami et al., 2025). Similarly, Zarghami et al., 2025 reported that nano-ZnO supplementation in high-yielding dairy cows improved animal performance and may serve as a cost-effective alternative to conventional ZnO supplementation. When performance variables were expressed relative to metabolic body weight (BW^0.75^), the positive responses remained consistent, indicating proportional improvements across body size. The observed reduction in ammonia concentration, together with improved growth performance, may also suggest greater efficiency of microbial protein utilization and nutrient partitioning toward productive functions rather than nitrogen excretion. Supporting this interpretation, Qu et al., 2023 reported that ZnO nanoparticles promoted microbial growth, microbial protein synthesis, and energy utilization efficiency during early ruminal incubation, while also improving intestinal morphology, including villus height and villus height-to-crypt depth ratio.

Although hematological parameters were not significantly affected overall, numerical trends were observed, including lower neutrophil, monocyte, and eosinophil counts and higher lymphocyte proportions. These trends could be associated with improved immune status or reduced physiological stress, although direct immunomodulatory mechanisms were not evaluated in the present meta-analysis. Wessels et al., 2017 suggested that Zn plays an important role in immune regulation through interactions with inflammatory mediators and zinc-binding proteins involved in host defense responses.

Regarding physiological response parameters, ALP activity increased, while AST and ALT remained unaffected. In addition, serum Zn concentration also showed toward an increase, suggesting improved Zn bioavailability following supplementation. The increase in ALP activity may reflect the physiological role of Zn in enzyme activation and mineral metabolism, as alkaline phosphatase is a Zn-dependent enzyme involved in phosphate metabolism, tissue growth, and bone mineralization (Bucci et al., 2014; Shu et al., 2022). However, because the present meta-analysis did not directly evaluate reproductive or bone metabolism parameters, these interpretations should be considered suggestive rather than confirmatory. Overall, the elevated serum Zn concentration indicates that supplemented Zn remained biologically available within systemic circulation.

The present findings are generally consistent with previous meta-analysis evaluating zinc supplementation in ruminants. Darabi et al., 2026 reported that dietary Zn supplementation improved dry matter intake, weight gain, ruminal total VFA concentration, and DM digestibility in lambs, with optimal responses occurring at approximately 28–42 mg/kg DM depending on the parameter evaluated. Similarly, Angeles-Hernandez et al., 2021 demonstrated that Zn supplementation significantly increased DMI and ADG while improving feed efficiency in small ruminants, with species, breed, Zn source, production level, and supplementation dosage identified as important sources of heterogeneity. In agreement with these findings, the present meta-analysis showed positive effects of ZnO supplementation on DMI, ADG, and total VFA concentration, while subgroup analysis indicated that animal species and Zn form contributed to variations in response.

However, unlike previous meta-analyses that primarily focused on growth performance and mineral metabolism, the present study additionally evaluated *in vitro* and *in vivo* fermentation responses and identified a significant reduction in methane emissions, suggesting that ZnO supplementation may contribute not only to improved animal performance but also to enhanced rumen fermentation efficiency and environmental sustainability. Nevertheless, the substantial heterogeneity observed across several variables indicates that the magnitude of the response to ZnO supplementation remains dependent on factors such as animal species, Zn source, dosage, and feeding conditions. Our meta-regression analysis further demonstrated that Zn dose significantly moderated ADG responses, suggesting that growth performance may be influenced by the level of Zn supplementation within the evaluated dose range. Although the relatively small number of available studies precluded a formal dose-response meta-analysis using restricted cubic spline models, this finding provides preliminary evidence of a dose-dependent relationship. Future meta-analyses incorporating a larger body of literature and a wider range of supplementation levels should explore potential non-linear dose-response patterns and threshold effects using flexible modelling approaches.

### Subgroup analysis on zinc oxide form

The subgroup analysis of this meta-analysis study indicates that the physicochemical form of ZnO plays a crucial role in modulating *in vitro* rumen fermentation parameters, particularly methane production and VFA profiles such as propionate. Statistically, nZnO and ZnO reduced methanogenesis. The particle size and specific surface area of ​​nZnO should provide higher reactivity than the ZnO conventional form (Anders et al., 2018). However, these data indicate that the effectiveness of nZnO in modulating *in vitro* fermentation varies widely. The greater efficacy of nZnO compared with conventional ZnO may primarily be attributed to its unique physicochemical characteristics, particularly its smaller particle size, larger surface area, and higher bioavailability (Sarker et al., 2018). Nanoparticles possess substantially greater reactive surface area per unit mass, enabling more extensive interactions with ruminal microbes, digestive substrates, and intestinal absorptive surfaces. This enhanced surface reactivity likely improves zinc solubility and facilitates more efficient penetration through biological membranes, thereby increasing zinc utilization efficiency compared with conventional ZnO. Consequently, nZnO may exert stronger modulatory effects on ruminal microbial activity and nutrient metabolism even at relatively low inclusion rates.

Conventional ZnO has relatively low solubility in the gastrointestinal tract, limiting zinc absorption and increasing mineral excretion. In contrast, nanoparticles can cross epithelial barriers more effectively through paracellular transport, endocytosis, or passive diffusion, thereby enhancing systemic zinc availability. According to Yang et al., 2025, zinc supplementation in animal feed is generally classified into organic and inorganic categories. Among the inorganic varieties, zinc oxide and zinc sulfate are frequently utilized. Zinc oxide is a white, odorless crystalline powder that, while insoluble in water, dissolves in acidic and sodium hydroxide solutions. It is valued for its contenthigh Zn density, which is roughly 72% elemental Zn.

### Subgroup analysis on species-based effects of zinc oxide

This meta-analysis showed that the effects of ZnO supplementation varied among ruminant species, especially in rumen fermentation and animal performance. In cattle and sheep, ZnO consistently reduced ruminal ammonia concentration, suggesting better nitrogen utilization and microbial protein synthesis. However, cattle showed a greater increase in total VFA concentration, whereas sheep had a larger improvement in DMD. These differences may be related to species-specific variation in rumen characteristics, such as rumen size, retention time, microbial population, and nutrient metabolism. The larger rumen capacity and longer feed retention time in cattle may support higher fermentative activity and VFA production, while the faster digesta passage and selective feeding behavior of sheep may enhance the response to micronutrient supplementation (Moyo et al., 2017). Even so, both species showed higher ADG without changes in DMI, indicating improved feed efficiency rather than increased feed consumption.

The physiological response to ZnO supplementation also differed among species. Most hematological and biochemical parameters remained stable in cattle and sheep, indicating that ZnO did not adversely affect physiological status. In goats, however, monocyte and eosinophil percentages tended to decrease, suggesting a potential immunomodulatory effect. Goats also appeared to exhibit greater changes in mineral status, particularly increased calcium and phosphate concentrations, which may reflect differences in mineral metabolism and absorption efficiency. Overall, the results suggest that ZnO supplementation can improve rumen efficiency and animal performance in ruminants, although the magnitude and nature of the response may vary according to species-specific physiological and metabolic characteristics. Nevertheless, an important limitation of the present subgroup analysis is the unequal distribution of studies among animal species. In particular, some goat-specific SMD was derived from fewer than three studies, resulting in wide confidence intervals and reduced statistical power. Therefore, the findings for goats should be considered exploratory and interpreted with caution until additional evidence becomes available.

Several limitations should be considered when interpreting the findings of this meta-analysis. First, substantial heterogeneity was observed for several outcome variables, indicating considerable variation among studies in animal species, ZnO form, supplementation level, dietary composition, and experimental duration. Second, the number of available studies was limited for several physiological and hematological parameters, which may have reduced the statistical power to detect treatment effects. Third, although significant associations between ZnO supplementation and fermentation or performance responses were identified, the biological mechanisms responsible for these effects could not be directly evaluated. Therefore, mechanistic interpretations should be regarded as potential explanations rather than definitive causal conclusions. Future studies integrating microbial, molecular, and metabolomic approaches may provide greater insight into the pathways through which ZnO influences rumen function and animal productivity.

## Conclusion

This meta-analysis provides the first integrated quantitative evaluation of ZnO supplementation on rumen fermentation and animal performance in ruminants. Overall, ZnO supplementation consistently shifted rumen fermentation toward more energetically efficient pathways, characterized by increased propionate production, reduced methanogenesis, and improved metabolizable energy, which translated into enhanced nitrogen utilization, higher total VFA production, dry matter digestibility, and improved average daily gain and feed intake. Comparative analysis shows that nZnO exhibited greater antimethanogenic efficacy than ZnO. Environmentally, ZnO supplementation offers dual benefits by mitigating enteric methane emissions while improving feed-energy utilization, supporting more sustainable ruminant production systems. However, these conclusions should be interpreted cautiously because substantial heterogeneity was observed across several outcomes, indicating that dietary composition, zinc dose, animal species, basal mineral status, and experimental duration strongly influenced response magnitude. Although the data c support positive effects of zinc supplementation, the magnitude and consistency of these responses remain context-dependent. Future studies should prioritize standardized dose–response experiments, harmonized methodological approaches, and microbiome-based investigations to clarify the mechanistic interactions between zinc supplementation, rumen microbial ecology, productive performance, and long-term environmental sustainability.

## Declaration of AI used

During the preparation of this manuscript, the authors used PaperPal to improve the English language and readability of the text. After using this tool, the authors reviewed and edited the content as needed and takes full responsibility for the final contents of the publication

## Statement for studies in humans/animals

This study was conducted using generated data from published experiments without directly used animals. Thus, it is not necessary to obtain approval from the Animal Ethics Committee of Universitas Sebelas Maret or related institution.

## CRediT authorship contribution statement

**Rahmat Hidayat:** Writing – review & editing, Supervision, Resources, Project administration, Methodology, Funding acquisition, Conceptualization. **Mardiah Rahmadani:** Writing – review & editing, Supervision, Resources, Project administration, Formal analysis, Conceptualization. **Fenda Alvionita Fhonna:** Writing – original draft, Validation, Investigation. **Laily Rinda Ardani:** Writing – original draft, Validation, Investigation. **Aldena Bina Salimah:** Writing – original draft, Validation, Investigation. **Ujang Hidayat Tanuwiria:** Writing – original draft, Validation, Investigation. **Adam Cieslak:** Writing – review & editing, Visualization, Validation, Supervision. **Malgorzata Szumacher-Strabel:** Writing – review & editing, Visualization, Validation, Supervision. **Zora Varadyova:** Writing – review & editing, Visualization, Validation, Supervision. **Anuraga Jayanegara:** Writing – review & editing, Visualization, Validation, Supervision. **Aeni Nurlatifah:** Writing – review & editing, Validation, Supervision. **Amlan Kumar Patra:** Writing – review & editing, Visualization, Validation, Supervision. **Agung Irawan:** Writing – review & editing, Visualization, Validation, Supervision. **Yulianri Rizki Yanza:** Writing – original draft, Methodology, Investigation, Formal analysis, Data curation.

## Declaration of competing interest

The authors declare that they have no known competing financial interests or personal relationships that could have appeared to influence the work reported in this paper.

## Data Availability

The dataset underpinning the results in the present study can be obtained from the corresponding author and will be made available upon reasonable request.

## Appendix Supplementary materials

Image, application 1

## References

[bib0001] Abdollahi M., Rezaei J., Fazaeli H. (2020). Performance, rumen fermentation, blood minerals, leukocyte and antioxidant capacity of young Holstein calves receiving high-surface ZnO instead of common ZnO. Archives of Animal Nutrition.

[bib0002] Alijani K., Rezaei J., Rouzbehan Y. (2020). Effect of nano-ZnO, compared to ZnO and Zn-methionine, on performance, nutrient status, rumen fermentation, blood enzymes, ferric reducing antioxidant power and immunoglobulin G in sheep. Animal Feed Science and Technology.

[bib0003] Anders C.B., Eixenberger J.E., Franco N.A., Hermann R.J., Rainey K.D., Chess J.J., Punnoose A., Wingett D.G. (2018). ZnO nanoparticle preparation route influences surface reactivity, dissolution and cytotoxicity. Environmental Science: Nano.

[bib0004] Angeles-Hernandez J.C., Miranda M., Muñoz-Benitez A.L., Vieyra-Alberto R., Morales-Aguilar N., Paz E.A., Gonzalez-Ronquillo M. (2021). Zinc supplementation improves growth performance in small ruminants: A systematic review and meta-regression analysis. Animal Production Science.

[bib0005] Arelovich H.M., Owens F.N., Horn G.W., Vizcarra J.A. (2000). Effects of supplemental zinc and manganese on ruminal fermentation, forage intake, and digestion by cattle fed prairie hay and urea. Journal of Animal Science.

[bib0006] Bucci D., Isani G., Giaretta E., Spinaci M., Tamanini C., Ferlizza E., Galeati G. (2014). Alkaline phosphatase in boar sperm function. Andrology.

[bib0007] Buck R.J., Fieberg J., Larkin D.J. (2022). The use of weighted averages of Hedges’ d in meta-analysis: Is it worth it?. Methods in Ecology and Evolution.

[bib61] Cai Z., Wang J., Zhang Y., Li X., Luo J., Gao X., Guo M. (2025). Zinc and animal health: An in-depth exploration of its role in physiological functions and regulatory molecular mechanisms. Journal of Animal Science and Biotechnology.

[bib0008] Darabi M., Hozhabri F., Piray A., Foroutanifar S. (2026). Zinc supplementation on growth performance, mineral metabolism and nutrient digestibility in lambs: A systematic review and dose–response meta-analysis. Biological Trace Element Research.

[bib59] DerSimonian R., Laird N. (1986). Meta-analysis in clinical trials. Controlled Clinical Trials.

[bib0009] EFSA Panel on Additives and Products or Substances used in Animal Feed (FEEDAP) (2012). Scientific opinion on safety and efficacy of zinc compounds (E6) as feed additive for all animal species: Zinc oxide, based on a dossier submitted by Grillo Zinkoxid GmbH/EMFEMA. EFSA Journal.

[bib0010] Fellner V., Durosoy S., Kromm V., Spears J.W. (2021). Effects of supplemental zinc on ruminal fermentation in continuous cultures. Applied Animal Science.

[bib0011] Geetha K., Chellapandian M., Arulnathan N., Ramanathan A. (2020). Nano zinc oxide – An alternate zinc supplement for livestock. Veterinary World.

[bib60] Higgins J.P.T., Thomas J., Chandler J., Cumpston M., Li T., Page M.J., Welch V.A. (2019). *Cochrane Handbook for Systematic Reviews of Interventions*.

[bib0012] Hosseini-Vardanjani S.F., Rezaei J., Karimi-Dehkordi S., Rouzbehan Y. (2020). Effect of feeding nano-ZnO on performance, rumen fermentation, leukocytes, antioxidant capacity, blood serum enzymes and minerals of ewes. Small Ruminant Research.

[bib0013] Irawan A., Azmi A.F.M., Rahman M.A., Yanza Y.R., Muchlas M., Abu Hassim H., Sofyan A., Jayanegara A., Gao M., Cieslak A., Niderkorn V., Ates S. (2026). Meta-analysis of the effects of dietary interventions to alleviate heat stress in lactating dairy cows. Journal of Dairy Science.

[bib0015] Jo Y.H., Kim W.S., Kim Y.R., Ju M.S., Nejad J.G., Lee H.G. (2024). Impacts of protein and energy levels on rumen fermentation and microbial activity under different incubation temperatures. Animals.

[bib0016] Kegley E.B., Ball J.J., Beck P.A. (2016). Impact of mineral and vitamin status on beef cattle immune function and health. Journal of Animal Science.

[bib0017] Khaksar V., Hervo F., Même N., Monteiro A.R., Narcy A. (2025). Effects of dietary calcium and microbial phytase on the bioavailability of two sources of zinc in broilers. British Poultry Science.

[bib0018] Kholif A.E., Anele A., Chahine M., Anele U.Y. (2025). Applications of nanotechnology in ruminant animal production: Advances, challenges, and future prospects. Nanomaterials.

[bib0019] Leško M., Bombárová A., Petrič D., Batťányi D., Komáromyová M., Königová A., Babják M., Halada Ľ., David S., Łukomska A., Pawlak P., Sidoruk P., Cieslak A., Čobanová K., Váradyová Z., Várady M. (2025). Effect of zinc oxide nanoparticle supplementation on parasite infection and rumen environment of grazing lambs. Frontiers in Veterinary Science.

[bib0021] Liberati A., Altman D.G., Tetzlaff J., Mulrow C., Gøtzsche P.C., Ioannidis J.P.A., Clarke M., Devereaux P.J., Kleijnen J., Moher D. (2009). The PRISMA statement for reporting systematic reviews and meta-analyses of studies that evaluate healthcare interventions: Explanation and elaboration. BMJ (Clinical research ed.).

[bib0022] Liu J., Ma F., Degen A., Sun P. (2023). The effects of zinc supplementation on growth, diarrhea, antioxidant capacity, and immune function in Holstein dairy calves. Animals.

[bib0023] Luo J., Wu X., Chen D., Yu B., He J. (2024). Dietary ferulic acid supplementation enhances antioxidant capacity and alleviates hepatocyte pyroptosis in diquat challenged piglets. Journal of Animal Science and Biotechnology.

[bib0024] Lou J., Xiang Z., Li J., Cui S., Huang N., Jin G., Le X., Fan Y., Sun Q. (2025). The beneficial impact of virtual reality in the burn wound care of pediatric patients: An updated systematic review and meta-analysis. Burns : Journal of the International Society for Burn Injuries.

[bib0026] Maqsood A., Khan Z.I., Ahmad K., Akhtar S., Ashfaq A., Malik I.S., Sultana R., Nadeem M., Alkahtani J., Dwiningsih Y., Elshikh M.S. (2022). Quantitative evaluation of zinc metal in meadows and ruminants for health assessment: Implications for humans. Environmental Science and Pollution Research.

[bib0028] Miller W.J., Wells E.S., Gentry R.P., Neathery M.W. (1971). Endogenous zinc excretion and 65 Zn metabolism in Holstein calves fed intermediate to high but nontoxic zinc levels in practical diets. The Journal of Nutrition.

[bib0029] Min B.R., Gurung N., Shange R., Solaiman S. (2019). Potential role of rumen microbiota in altering average daily gain and feed efficiency in meat goats fed simple and mixed pastures using bacterial tag-encoded FLX amplicon pyrosequencing. Journal of Animal Science.

[bib0030] Moyo M., Gueguim Kana E.B., Nsahlai I.V. (2017). Modelling of digesta passage rates in grazing and browsing domestic and wild ruminant herbivores. South African Journal of Animal Science.

[bib0031] Najafzadeh H., Ghoreishi S.M., Mohammadian B., Rahimi E., Afzalzadeh M.R., Kazemivarnamkhasti M., Ganjealidarani H. (2013). Serum biochemical and histopathological changes in liver and kidney in lambs after zinc oxide nanoparticles administration. Veterinary World.

[bib0032] Neathery M.W., Miller W.P., Blackmon D.M., Gentry R.P., Jones J.B. (1973). Absorption and tissue zinc content in lactating dairy cows as affected by low dietary zinc. Journal of Animal Science.

[bib0033] Petrič D., Mikulová K., Bombárová A., Batťányi D., Čobanová K., Kopel P., Łukomska A., Pawlak P., Sidoruk P., Kotwica S., Cieslak A., Váradyová Z. (2024). Afficacy of zinc nanoparticle supplementation on ruminal environment in lambs. BMC Veterinary Research.

[bib0034] Puchala R., Sahlu T., Davis J.J. (1999). Effects of zinc-methionine on performance of Angora goats. Small Ruminant Research.

[bib0035] Qu J., Zuo X., Xu Q., Li M., Zou L., Tao R., Liu X., Wang X., Wang J., Wen L. (2023). Effect of two particle sizes of nano zinc oxide on growth performance, immune function, digestive tract morphology, and intestinal microbiota composition in broilers. Animals.

[bib0036] Rahmadani M., Tryas A.A., Susanto I., Nahrowi N., Khotijah L., Jayanegara A. (2024). Enhancing resistant starch in foods through organic acid intervention: A meta-analysis on thermal properties, nutrient composition, and *in vitro* starch digestibility. Journal of Agriculture and Food Research.

[bib0037] Riazi H., Rezaei J., Rouzbehan Y. (2019). Effects of supplementary nano-ZnO on *in vitro* ruminal fermentation, methane release, antioxidants, and microbial biomass. Turkish Journal of Veterinary and Animal Sciences.

[bib0038] Rodríguez-Maya M.A., Domínguez-Vara I.A., Trujillo-Gutiérrez D., Morales-Almaráz E., Sánchez-Torres J.E., Bórquez-Gastelum J.L., Acosta-Dibarrat J., Grageola-Nuñez F., Rodríguez-Carpena J.G. (2019). Growth performance parameters, carcass traits, and meat quality of lambs supplemented with zinc methionine and (Or) zinc oxide in feedlot system. Canadian Journal of Animal Science.

[bib0040] Sanchez-Meca J., Marin-Martinez (2010). Meta analysis, international encyclopedia of education.

[bib0039] Sarker N.C., Keomanivong F., Borhan M., Rahman S., Swanson K. (2018). *In vitro* evaluation of nano zinc oxide (nZnO) on mitigation of gaseous emissions. Journal of Animal Science and Technology.

[bib62] Sadegzadeh-Sadat M., Anassori E., Khalilvandi-Behroozyar H., Asri-Rezaei S. (2021). The effects of Zinc-Methionine on glucose metabolism and insulin resistance during late pregnancy in ewes. Domestic Animal Endocrinology.

[bib0041] Shi L., Lin L. (2019). The trim-and-fill method for publication bias: Practical guidelines and recommendations based on a large database of meta-analysis. Medicine (Baltimore).

[bib0042] Shu J., Tan A., Li Y., Huang H., Tang J. (2022). The correlation between serum total alkaline phosphatase and bone mineral density in young adults. BMC Musculoskeletal Disorders.

[bib0043] Sterne J.A.C., Savović J., Page M.J., Elbers R.G., Blencowe N.S., Boutron I., Higgins J.P.T. (2019). RoB 2 : A revised tool for assessing risk of bias in randomised trials. BMJ.

[bib0045] Tseten T., Sanjorjo R.A., Kwon M., Kim S.W. (2022). Strategies to mitigate enteric methane emissions from ruminant animals. Journal of Microbiology and Biotechnology.

[bib0046] Vigh A., Criste A., Gragnic K., Moquet L., Gerard C. (2023). Ruminal solubility and bioavailability of inorganic trace mineral sources and effects on fermentation activity measured *in vitro*. Agriculture.

[bib0047] Wang C., Zhang L., Su W., Ying Z., He J., Zhang L., Ma Y. (2017). Zinc oxide nanoparticles as a substitute for zinc oxide or colistin sulfate: Effects on growth, serum enzymes, zinc deposition, intestinal morphology and epithelial barrier in weaned piglets. PLOS ONE.

[bib0048] Wessels I., Maywald M., Rink L. (2017). Zinc as a gatekeeper of immune function. Nutrients.

[bib0049] Xie S., Ying Z., Xiu Z., Sun Y., Yang Q., Gao H., Fan W., Wu Y. (2024). Zinc oxide nanoparticles improve lactation and metabolism in dairy goats by modulating the rumen microbiota. Frontiers in Microbiology.

[bib0050] Yang J., Xiong D., Long M. (2025). Zinc oxide nanoparticles as next-generation feed additives: Bridging antimicrobial efficacy, growth promotion, and sustainable strategies in animal nutrition. Nanomaterials.

[bib0051] Yatoo M.I., Saxena A., Deepa P.M., Habeab B.P., Devi S., Jatav R.S., Dimri U. (2013). Role of trace elements in animals: A review. Veterinary World.

[bib0052] Youssef A.M., Azzaz H.H., Hassaan N.A., El-Nomeery Y.A., Shakweer W.M.E. (2022). Facile green preparation of zinc oxide nanoparticles and its effects on in-vitro feed degradation, ruminal fermentation, and total gas production. Egyptian Journal of Chemistry.

[bib0053] Yusof M.H., Mohamad R., Zaidan U.H., Abdul Rahman N.A. (2019). Microbial synthesis of zinc oxide nanoparticles and their potential application as an antimicrobial agent and a feed supplement in animal industry: A review. Journal of Animal Science and Biotechnology.

[bib0054] Yusuf A., Akinyemi B., Surajudeen M., Sowande O. (2025). Influence of nano zinc supplementation on the haematological properties, serum biochemical constituents, rumen microbes and minerals of West African dwarf sheep. Iranian Journal of Applied Animal Science.

[bib0055] Yusuf A.O., Adeyi T.K., Oni A.O., Owalabi A.J., Sowande S.O. (2023). Nano zinc supplementation in ruminant’s livestock, influence on physiological, immune functions and oxidative stability of West African dwarf goat bucks. Comparative Clinical Pathology.

[bib0056] Zarghami A., Ganjkhanlou M., Zali A., Fekri A., Palangi V. (2025). Effects of nano-zinc oxide supplementation on milk yield, rumen fermentation, nutrient digestibility, and blood indices of high-yielding dairy cows. Frontiers in Veterinary Science.

[bib0057] Zhang H., Guan W., Li L., Guo D., Zhang X., Guan J., Luo R., Zheng S., Fu J., Cheng Y., He Q. (2022). Dietary carbon loaded with nano-ZnO alters the gut microbiota community to mediate bile acid metabolism and potentiate intestinal immune function in fattening beef cattle. BMC Veterinary Research.

[bib0058] Zhang Y., Li X., Li J., Khan M.Z.H., Ma F., Liu X. (2021). A novel zinc complex with antibacterial and antioxidant activity. BMC Chemistry.

