# Supplementary material for: The influence of zinc oxide utilization in ruminants: A systematic review and meta-analysis
Source: Vet Anim Sci. 2026 Aug 1;34:100796. doi: 10.1016/j.vas.2026.100796 (PMC13448448; doi:10.1016/j.vas.2026.100796)
Supplement: Supplementary file 1 [file mmc1.docx]

**Supplementary file**

**Moderators’ analysis**

Table S1. Moderators analyses of the dataset from *in vitro* experiment

| **Outcome** | **N** | **Moderator** | **Intercept** | **P-value** | **QE** | **QE P-value** | **Tau²** |
| --- | --- | --- | --- | --- | --- | --- | --- |
| pH | 19 | Zn form | 0.93 | 0.334 | 160.41 | 0.000 | 1.65 |
| pH |  | Animal species | 0.84 | 0.358 | 162.08 | 0.000 | 1.79 |
| pH |  | Breed | 17.25 | 0.000 | 144.49 | 0.000 | 0.02 |
| pH |  | Type of animal | 16.46 | 0.000 | 145.75 | 0.000 | 0.02 |
| Acetate | 13 | Zn form | 1.39 | 0.238 | 4.40 | 0.957 | 3.01 |
| Acetate |  | Animal species | 0.42 | 0.515 | 5.33 | 0.914 | 0.00 |
| Acetate |  | Breed | 0.42 | 0.515 | 5.33 | 0.914 | 0.00 |
| Propionate | 12 | Animal species | 0.50 | 0.478 | 2.78 | 0.734 | 54.50 |
| Propionate |  | Breed | 0.50 | 0.478 | 2.78 | 0.734 | 54.50 |
| Total VFA | 13 | Zn form | 0.83 | 0.363 | 12.49 | 0.488 | 35.10 |
| Total VFA |  | Animal species | 1.03 | 0.309 | 11.20 | 0.594 | 26.41 |
| Total VFA |  | Breed | 1.56 | 0.458 | 10.06 | 0.611 | 31.42 |
| Total VFA |  | Type of animal | 0.01 | 0.942 | 13.65 | 0.399 | 43.24 |
| NH_3_ | 15 | Zn form | 0.13 | 0.716 | 24.22 | 0.001 | 406.53 |
| NH_3_ |  | Animal species | 25.93 | 0.000 | 3.89 | 0.792 | 0.00 |
| NH_3_ |  | Breed | 25.93 | 0.000 | 3.89 | 0.792 | 0.00 |
| NH_3_ |  | Type of animal | 25.93 | 0.000 | 3.89 | 0.792 | 0.00 |

VFA = volatile fatty acid, Zn form = effect of different Zn form (ZnO, nZnO), Animal species = effect of animal species (cattle, dairy cows, sheep, goat), Type of animal = effect of difference types of animal (meat or milk producing animals).

Table S2. Moderators analyses of the dataset from *in vivo* experiment

| **Outcome** | **N** | **Moderator** | **Intercept** | **P-value** | **QE** | **QE_Pvalue** | **Tau^2^** |
| --- | --- | --- | --- | --- | --- | --- | --- |
| ADG | 8 | Zn form | 1.51 | 0.219 | 4.45 | 0.616 | 0.00 |
| ADG |  | Zn doses | 4.23 | 0.040 | 1.73 | 0.943 | 0.00 |
| ADG |  | Animal species | 5.10 | 0.078 | 0.86 | 0.973 | 0.00 |
| ADG |  | Breed | 5.41 | 0.248 | 0.32 | 0.572 | 0.00 |
| ADG |  | Type of animal | 0.28 | 0.598 | 5.36 | 0.499 | 73.56 |
| DMI | 9 | Zn form | 1.13 | 0.288 | 20.59 | 0.004 | 13.26 |
| DMI |  | Zn doses | 0.02 | 0.875 | 20.57 | 0.004 | 13.50 |
| DMI |  | Animal species | 0.84 | 0.659 | 12.57 | 0.050 | 15.23 |
| DMI |  | Breed | 15.47 | 0.004 | 2.91 | 0.233 | 0.00 |
| DMI |  | Type of animal | 0.30 | 0.582 | 16.75 | 0.019 | 12.93 |
| pH | 17 | Zn form | 0.04 | 0.841 | 48.22 | 0.000 | 3.53 |
| pH |  | Zn doses | 0.00 | 0.957 | 47.54 | 0.000 | 3.86 |
| pH |  | Animal species | 0.37 | 0.544 | 47.58 | 0.000 | 4.41 |
| pH |  | Breed | 1.34 | 0.854 | 21.87 | 0.005 | 0.00 |
| pH |  | Type of animal | 0.37 | 0.544 | 47.58 | 0.000 | 4.41 |
| Acetate | 11 | Zn form | 0.07 | 0.797 | 3.28 | 0.952 | 0.00 |
| Acetate |  | Zn doses | 0.70 | 0.403 | 2.65 | 0.977 | 0.00 |
| Acetate |  | Animal species | 1.77 | 0.183 | 1.57 | 0.997 | 0.00 |
| Acetate |  | Breed | 2.42 | 0.490 | 0.63 | 0.987 | 0.00 |
| Acetate |  | Type of animal | 1.77 | 0.183 | 1.57 | 0.997 | 0.00 |
| Propionate | 11 | Zn form | 1.03 | 0.309 | 4.24 | 0.895 | 0.00 |
| Propionate |  | Zn doses | 0.37 | 0.541 | 4.90 | 0.843 | 0.00 |
| Propionate |  | Animal species | 1.16 | 0.281 | 4.11 | 0.904 | 0.00 |
| Propionate |  | Breed | 2.18 | 0.536 | 1.56 | 0.906 | 0.00 |
| Propionate |  | Type of animal | 1.16 | 0.281 | 4.11 | 0.904 | 0.00 |
| Total VFA | 11 | Zn form | 1.45 | 0.229 | 2.93 | 0.967 | 0.00 |
| Total VFA |  | Zn doses | 0.87 | 0.352 | 3.51 | 0.941 | 0.00 |
| Total VFA |  | Animal species | 0.00 | 0.983 | 4.38 | 0.885 | 0.00 |
| Total VFA |  | Breed | 2.27 | 0.517 | 2.00 | 0.849 | 0.00 |
| Total VFA |  | Type of animal | 0.00 | 0.983 | 4.38 | 0.885 | 0.00 |
| NH_3_ | 11 | Zn form | 0.76 | 0.385 | 11.50 | 0.243 | 67.85 |
| NH_3_ |  | Zn doses | 0.15 | 0.697 | 11.81 | 0.224 | 82.93 |
| NH_3_ |  | Animal species | 0.07 | 0.798 | 11.60 | 0.237 | 115.73 |
| NH_3_ |  | Breed | 5.56 | 0.135 | 2.18 | 0.824 | 0.00 |
| NH_3_ |  | Type of animal | 0.07 | 0.798 | 11.60 | 0.237 | 115.73 |

ADG = average daily gain, DMI = dry matter intake, VFA = volatile fatty acid, Zn form = effect of different Zn form (ZnO, nZnO), Zn doses = effect of Zn level in the diet (continuous data), Animal species = effect of animal species (cattle, dairy cows, sheep, goat), Type of animal = effect of difference types of animal (meat or milk producing animals).
